# Temperature, sediment resuspension, and salinity drive the prevalence of *Vibrio vulnificus* in the coastal Baltic Sea

**DOI:** 10.1128/mbio.01569-24

**Published:** 2024-09-19

**Authors:** Víctor Fernández-Juárez, David J. Riedinger, Joao Bosco Gusmao, Luis Fernando Delgado-Zambrano, Guillem Coll-García, Vasiliki Papazachariou, Daniel P. R. Herlemann, Christian Pansch, Anders F. Andersson, Matthias Labrenz, Lasse Riemann

**Affiliations:** 1 Marine Biological Section, Department of Biology, University of Copenhagen, Copenhagen, Denmark; 2 Department of Biological Oceanography, Leibniz Institute for Baltic Sea Research Warnemünde (IOW), Rostock, Germany; 3 Environmental and Marine Biology, Åbo Akademi University, Turku, Finland; 4 Science for Life Laboratory, Department of Gene Technology, KTH Royal Institute of Technology, Stockholm, Sweden; 5 Microbiology, Biology Department, University of the Balearic Islands, Palma de Mallorca, Spain; 6 Environmental Microbiology Group, Mediterranean Institute for Advanced Studies (CSIC-UIB), Esporles, Spain; 7 Estonian University of Life Sciences, Tartu, Estonia; Oregon State University, Corvallis, Oregon, USA

**Keywords:** Baltic Sea, pathogens, *Vibrio *spp., *Vibrio vulnificus*, sea surface temperature, salinity, sediment resuspension, phosphate, cyanobacteria

## Abstract

The number of *Vibrio*-related infections in humans, e.g., by *Vibrio vulnificus*, has increased along the coasts of the Baltic Sea. Due to climate change, vibriosis risk is expected to increase. It is, therefore, pertinent to design a strategy for mitigation of the vibriosis threat in the Baltic Sea area, but a prerequisite is to identify the environmental conditions promoting the occurrence of pathogenic *Vibrio* spp., like *V. vulnificus*. To address this, we sampled three coastal Baltic sites in Finland, Germany, and Denmark with salinities between 6 and 21 from May to October 2022. The absolute and relative abundances of *Vibrio* spp. and *V. vulnificus* in water were compared to environmental conditions, including the presence of the eelgrass *Zostera marina*, which has been suggested to reduce pathogenic *Vibrio* species abundance. In the water column, *V. vulnificus* only occurred at the German station between July and August at salinity 8.1–11.2. Temperature and phosphate (PO_4_^3–^) were identified as the most influencing factors for *Vibrio* spp. and *V. vulnificus*. The accumulation of *Vibrio* spp. in the sediment and the co-occurrence with sediment bacteria in the water column indicate that sediment resuspension contributed to *V. vulnificus* abundance. Interestingly, *V. vulnificus* co-occurred with specific cyanobacteria taxa, as well as specific bacteria associated with cyanobacteria. Although we found no reduction in *Vibrio* spp. or *V. vulnificus* associated with eelgrass beds, our study underscores the importance of extended heatwaves and sediment resuspension, which may elevate the availability of PO_4_^3–^, for *Vibrio* species levels at intermediate salinities in the Baltic Sea.

**IMPORTANCE:**

Elevated sea surface temperatures are increasing the prevalence of pathogenic *Vibrio* at higher latitudes. The recent increase in *Vibrio*-related wound infections and deaths along the Baltic coasts is, therefore, of serious health concern. We used culture-independent data generated from three Baltic coastal sites in Denmark, Germany, and Finland from May to October (2022), with a special focus on *Vibrio vulnificus*, and combined it with environmental data. Our temporal model shows that temperature, combined with sediment resuspension, drives the prevalence of *V. vulnificus* at intermediate salinities in the coastal Baltic Sea.

## INTRODUCTION

There are more than 130 recognized *Vibrio* spp. (https://www.bacterio.net/genus/vibrio), with approximately a dozen of them being associated with human illness, e.g., *Vibrio alginolyticus*, *Vibrio cholerae*, *Vibrio parahaemolyticus*, and *Vibrio vulnificus*. During recent years, the prevalence of non-cholera *Vibrio*-related wound infections and deaths has increased dramatically at higher latitudes, particularly along the brackish Baltic Sea’s salinity gradient that goes from 2 to 25 (1–3). Many of these cases are caused by *V. vulnificus*, commonly known as a flesh-eating bacterium, infecting open wounds and causing septicemia and necrosis, with a mortality rate of 25%, primarily among elderly, i.e., >65 years of age (1, 4). In the Nordic and Baltic countries, more than 1,055 cases of vibriosis, including *V. vulnificus*, were reported between 2014 and 2018, with most cases occurring during the summer (1, 5, 6), underscoring that vibriosis is a significant public health challenge in the region and that enhanced preventive measures are needed.

*Vibrio* spp. are commonly found free-living in the water column and in association with seagrasses, plankton, and aquatic animals (7). *Vibrio* spp. can also be frequently found in biofilms on sediment-trapped particles, which offer nutrient-rich environments (8–10). Seagrass meadows may play a role in promoting the sedimentation of particles and stabilizing the sediment through the reduction of turbulent flow, consequently reducing *Vibrio* species abundances in the water column (11, 12), but data are sparse. *Vibrio* species distribution exhibits strong seasonality, influenced by factors such as temperature, salinity, eutrophication, phytoplankton biomass, and turbidity (7, 13–17). The pathogen *V. vulnificus* thrives in brackish waters with salinities ranging from 5 to 25, and temperature is suggested to be the most influential factor affecting its presence and proliferation (15, 16). The increase in Baltic Sea surface temperatures is more than three times higher than the global ocean warming average (18), and global warming will conceivably stimulate the proliferation of pathogenic *Vibrio* spp., e.g., *V. vulnificus*, in the region, with a significant risk of proliferation occurring above 20°C (19).

Therefore, the pathogenic “*Vibrio* problem” in the Baltic Sea is predicted to be exacerbated by future climate change (20–22). From a public health perspective, there is a pertinent need for identifying the environmental conditions promoting outbreaks of pathogenic *Vibrio* spp., e.g., *V. vulnificus*, and ultimately to determine a strategy for mitigation of the vibriosis threat in the Baltic Sea area. In this study, we used culture-independent data generated from three Baltic coastal sites in Denmark, Germany, and Finland (from within to outside of eelgrass meadows, the water column, and sediment) with contrasting salinity and nutrient levels from May to October (2022) to determine drivers of *Vibrio* spp., with a special focus on the pathogen *V. vulnificus*.

## RESULTS

### Temporal dynamics of environmental and biological factors

**Fig 1:**
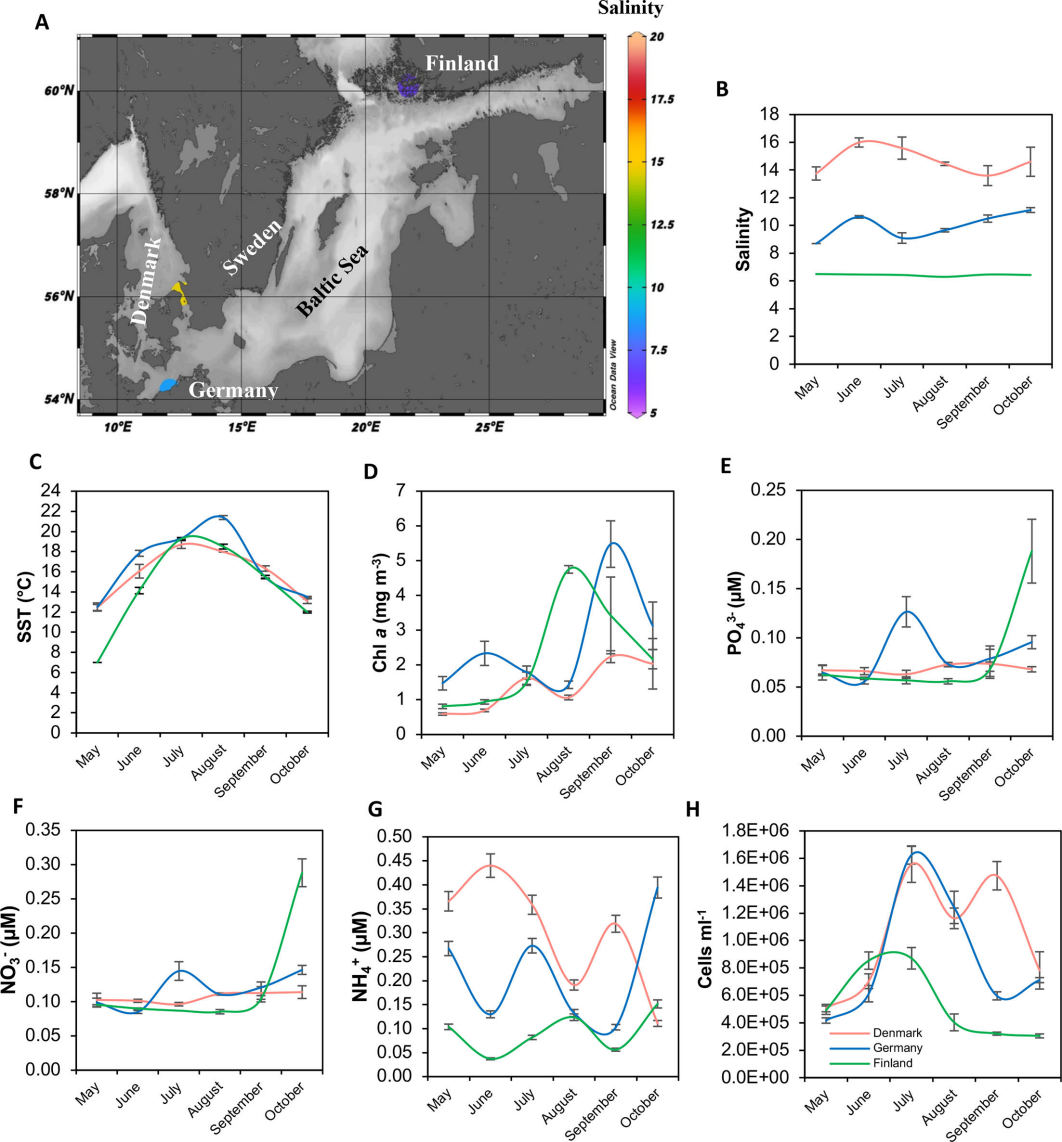
Sampling sites and temporal dynamics of selected parameters in the water column. (A) Map showing sampling stations in Denmark, Germany, and Finland. Seasonal dynamics of (B) salinity, (C) sea surface temperature (SST, °C), (D) chlorophyll *a* (Chl *a*, mg m^−3^), (E) PO_4_^3−^ (µM), (F) NO_3_^−^ (µM), (G) NH_4_^+^ (µM), and (H) bacterial abundance (cells mL^−1^). Values are the average of the nine replicates from the substations (“A”, “B”, and “C”) ± the standard error between the replicates. The average data from the two monthly sampling points in Denmark and Germany (July and August) are presented to enable comparisons with the Finnish station. In (A), the color codes represent the average value in the water column across the sampling period. Detailed information about the stations and substations is provided in Table S1.

From May to October 2022, we conducted monthly sampling along the Baltic Sea salinity gradient in Denmark (13–21.3), Germany (8–11.2), and Finland (6.3–6.5) (Table 1; Fig. 1A and B). The sea surface temperature (SST) ranged from 7 to 21.5°C (Fig. 1C). The highest Chl *a* concentration of about 5 mg m^−3^ was observed in late summer and fall in Finland and Germany (Fig. 1D). Phosphate (PO_4_^3−^) and nitrate (NO_3_^−^) exhibited a consistent pattern, both peaking during July and August at the German station, with concentrations reaching 0.13 µM and 0.14 µM, respectively (Fig. 1E and F). On the other hand, ammonia (NH_4_^+^) displayed a west-east gradient, reaching a maximum concentration of 0.3 µM (Fig. 1G). The highest bacterial abundance was from July to September in Denmark and Germany (ca. 10^6^ cells mL^−1^; Fig. 1H). All the water metadata are provided in Table S1.

**TABLE 1:** Sampling site characteristics from May to October 2022^*a*^

Sampling site and ID	Latitude (N)	Longitude (E)	Salinity range	SST range (C°)	Collection date	Sampling points	Sampling depth (m)
Julebæk, Denmark	56.05	12.57	13.1–21.3	11.8–19.5	May to October	12	1–2.5
Warnemünde, Germany	54.17	12.10	8.1–11.2	11.5–21.4	May to October	8	1–1.5
Ängsö Bay, Finland	60.10	21.71	6.3–6.5	7–19.3	May to October	6	2–4

^*a*^ Samples were collected no more than 200 m from the coast. In Denmark, the sampling was conducted twice per month, whereas in Germany and Finland, it was carried out once per month, except for July and August, where two samplings were conducted at the German station.

### *Vibrio* species composition and diversity in the Baltic Sea

**Fig 2:**
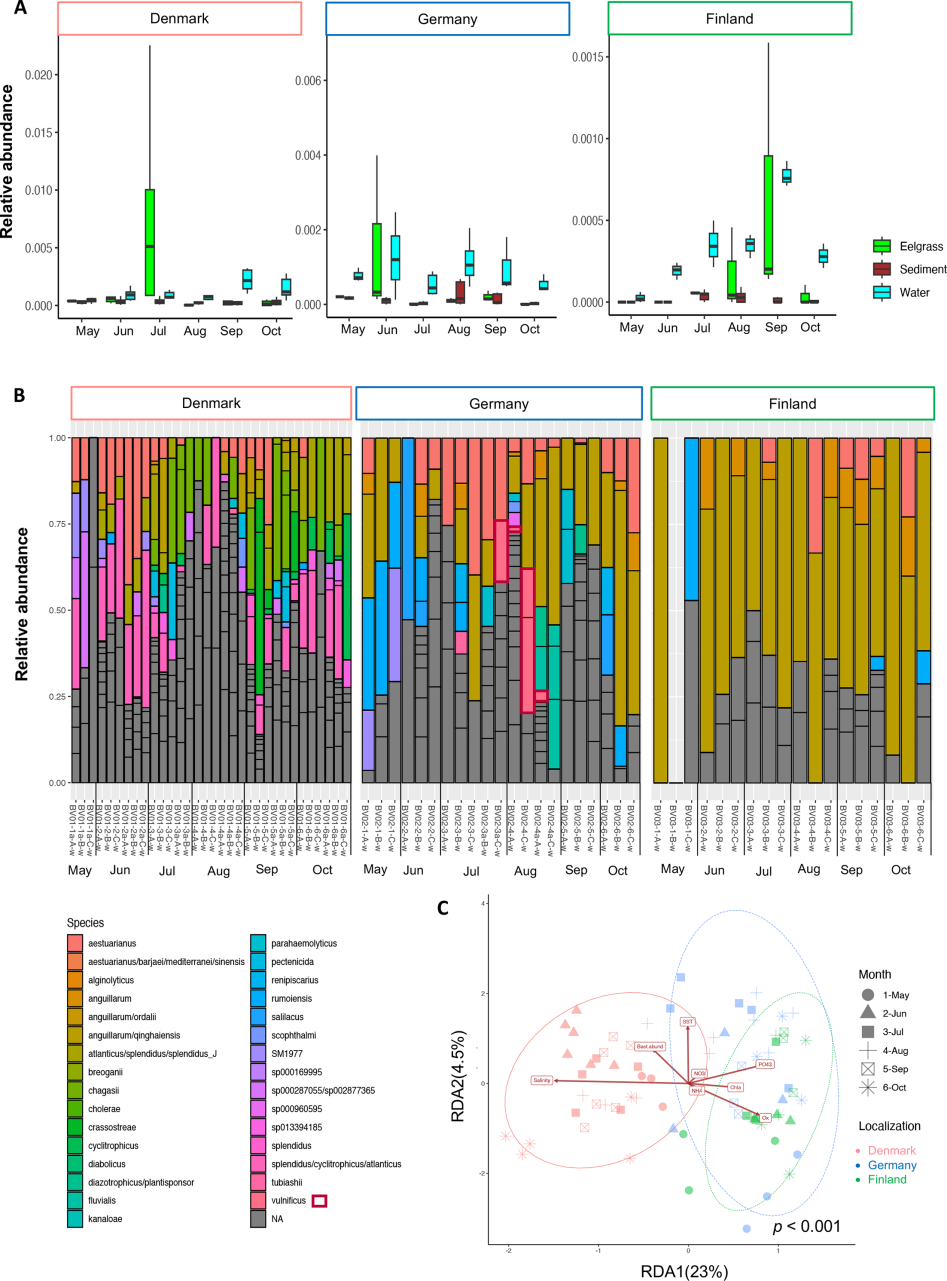
*Vibrio* community composition and dynamics from May to October (2022) in water and sediment at the Danish, German, and Finnish stations. (**A**) Relative abundance of *Vibrio* spp. on the water column, sediment, and eelgrass microbial population through the season. Values are the median, and vertical black lines indicate the position of the lower and upper quartiles of all the values collected in the three substations during the hole campaign in eelgrass, sediment, and water, and in the water column. (**B**) *Vibrio* communities in the water column. Sample ID clustered by month is shown on the plot. Two monthly sampling points were made in Denmark and Germany (July and August), and they are represented by an “a.” Within each color, each segment represents the relative abundance of ASVs having the same taxonomy. NA represents *Vibrio* spp. that could not be annotated to species level. *V. vulnificus* is highlighted in the plot by red squares. (**C**) RDA-constrained ordination plot of the *Vibrio* species community in the water column. Salinity, SST (°C), oxygen (Ox, mg L^−1^), PO_4_^3−^ (µM), NH_4_^+^ (µM), NO_3_^−^ (µM), and bacterial abundance (cells mL^−1^) were chosen as constraint variables. Eigenvalues are 17.09 for RDA1 and 4.02 for RDA2. The significance of the RDA model was assessed by an ANOVA-like permutation test, and ellipses enclose sample groups (Denmark, Germany, and Finland, *n* = 75). For (**C**), clr-transformed data were used to perform the analysis.

Based on Illumina sequencing, we generated a total of 143,935 amplicon sequence variants (ASVs) from 75 water, 204 sediment, and 78 eelgrass samples collected from May to October 2022. Importantly, triplicate DNA water samples from each substation were combined, resulting in one DNA water sample sequenced per substation and sampling point. The relative abundance of *Vibrio* spp. seemed to increase in Finland only during summer (Fig. 2A). There was no notable enrichment in relative abundance of *Vibrio* spp. in sediment and eelgrass, in comparison to water samples where *Vibrio* spp. accounted for an average of 0.03% of the total microbial community (Fig. 2A). The Shannon diversity of *Vibrio* spp. was higher in the water compared to the eelgrass and sediment environments (Fig. S1A). Notably, there was a significant difference in *Vibrio* spp. composition between water and sediment samples (Fig. S1B).

The 236 ASVs assigned as *Vibrio* spp. in the water column were annotated into 31 different species (Fig. 2B). The *Vibrio* community in the water differed between stations and changed over the season (Fig. 2B and C; *P* < 0.001). Indeed, the *Vibrio* species Shannon diversity increased across all stations during the summer season, with the Finnish station having the lowest salinity and the lowest diversity (Fig. S1C). Over the season, in Denmark, the *Vibrio* community was dominated by “ASV 6,774” (*Vibrio cyclitrophicus*), “ASV 7,595” (*Vibrio atlanticus* or *Vibrio splendidus*), and “ASV 7,725” (*Vibrio chagasii*), whereas “ASV 1,107” (*Vibrio anguillarum* or *Vibrio ordalii*) and “ASV 14,013” (*V. anguillarum*) dominated in Germany and Finland (Fig. 2B). These ASVs accounted for ca. 40% of the difference in *Vibrio* communities between Denmark, Finland, and Germany (Fig. 2C; SIMPER analysis). SST, salinity, inorganic nutrients (PO_4_^3–^, NO_3_^–^, and NH_4_^+^), Chl *a*, bacterial abundance, and oxygen explained 24% of the changes in the *Vibrio* community over the season (Fig. 2C; F = 2.45, *P* < 0.001). The “ASV 38,490” and “ASV 30,041,” annotated as *V. vulnificus*, were detected between July and August in Germany and accounted for an average of 5% and up to 50% of the *Vibrio* community (approximately 0.01% of the total microbial community). However, no ASVs affiliated with *V. vulnificus* were detected in Denmark or Finland.

### Temporal dynamics of *Vibrio* spp. and *V. vulnificus*

**Fig 3:**
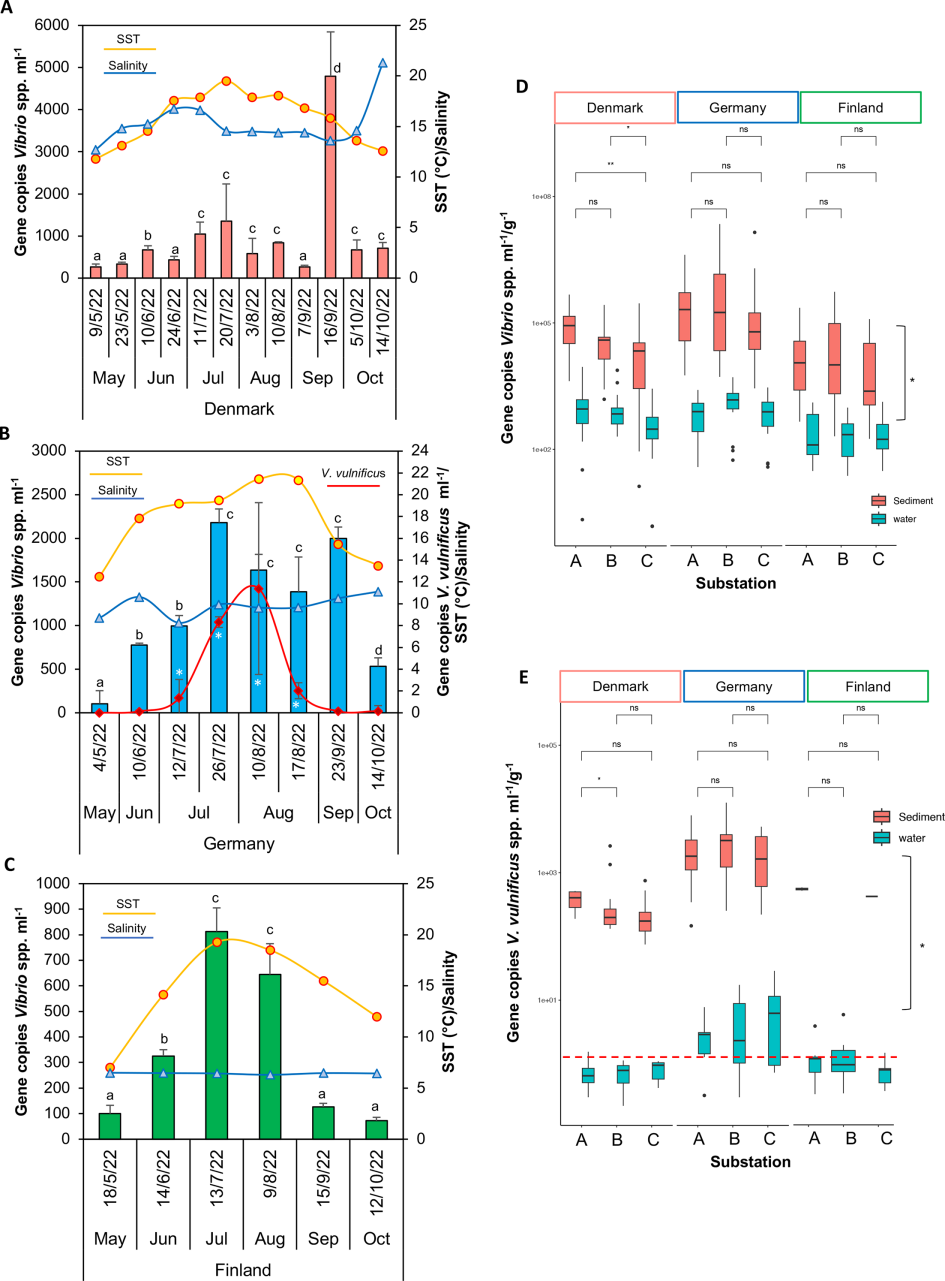
Dynamics of *Vibrio* species abundance in the water column at the three stations (**A**) Denmark, (**B**) Germany, and (**C**) Finland from May to October 2022. Abundances were proxied by ddPCR gene quantification*,* and *V. vulnificus* was only above the detection limit at the German station. Bars indicate *Vibrio* species abundance; red line indicates *V. vulnificus* abundance; orange lines indicate SST (yellow fill in [**B**] indicates the temperatures at which *V. vulnificus* was detected]; and blue lines indicate salinity. Values are average ± standard error (*n* = 9, i.e., combining the triplicates by substation). Comparison of gene copies of *Vibrio* spp. (**D**) and *V. vulnificus* (**E**) in water and sediment samples obtained from the substations; within (“A”), edge of (“B,” 15 m), and far from (“C,” 100 m) the eelgrass. Data from all the time points at the specific substation in Denmark (*n* = 36), Germany (*n* = 24), and Finland (*n* = 18) were combined. Values are the median, and vertical black lines indicate the position of the lower and upper quartiles. In (**E**), red lines indicate gene copies are under the detection limit. In (**A**), (**B**), and (**C**), letters show pairwise analysis among the variables (i.e., month) at each station, and in (**D**) and (**E**), asterisks indicate pairwise significant differences between substations, i.e., “A,” “B,” and “C,” or type of sample, i.e., water and sediment (*P < 0.05, **P < 0.01, ns [not significant] = *P* > 0.05), using a post hoc test (Wilcoxon) after a Kruskal–Wallis test.

Gene copies per milliliter seawater, i.e., 16S rRNA (*Vibrio* spp.) and *vvhA* (*V. vulnificus*), were quantified as a proxy for the prevalence of *Vibrio* spp. and *V. vulnificus*. They varied significantly over the season (Fig. 3A through C, *P* < 0.05) and correlated with the ASV abundance—calculated from the relative abundance multiplied by total bacterial abundance (cells mL^−1^) (23)—(r^2^ = 0.61 and 0.63, respectively; Fig. S2A and B). In Denmark, *Vibrio* spp. peaked in July (up to 1,000 copies mL^−1^) and September (up to 5,000 copies mL^−1^, Fig. 3A). In Germany, the highest abundance of *Vibrio* spp. was observed from July to September, reaching ca. 2,000 copies mL^−1^ (Fig. 3B). In Finland, *Vibrio* spp. reached their peak levels in July and August, but only reached 800 copies/mL (Fig. 3C). Importantly, and corroborating the 16S rRNA gene sequencing results, *V. vulnificus* was only quantifiable in Germany, peaking in summer with up to 30 copies mL^−1^ when SST values ranged from 19.2°C to 21.4°C (Fig. 3B), whereas it was below the detection limit at the Danish and Finish stations.

The sampling design was based on earlier findings suggesting a negative impact of *Z. marina* on *Vibrio* spp., including pathogenic species (12). However, in our study, in Denmark, substations “A” and “B,” located in the middle of the eelgrass bed and 15 m away, respectively, had higher levels of *Vibrio* spp. compared to substation “C,” located 100 m away from the eelgrass, both in the water column and sediment (Fig. 3D). In contrast, no discernible impact was observed on *V. vulnificus* in the water column (i.e., in the German station) (Fig. 3E).

The abundance of *Vibrio* spp. and *V. vulnificus* genes were 100- and 1,000-fold higher, respectively, in the sediment than in the water (per weight [gram] vs per volume [milliliter]; Fig. 3D and E), and abundances in sediments correlated with abundances in water (Table 2). Sediment composition differed between stations, where the German sediment contained slightly more fine materials, i.e., clay and silt (Table S2), and the highest *Vibrio* species abundance (>10^6^ gene copies g^−1^ during the summer months, i.e., 10 times more than in Denmark and Finland, Fig. 3D; Fig. S3A through C). *V. vulnificus* had the highest abundance in Germany, with >10^3^ gene copies g^−1^ sediment in August. It was also detectable in Denmark, with up to 10^2^ gene copies g^−1^ sediment, but was absent in Finland (Fig. S3A and B). Interestingly, in the sediment, *V. vulnificus* remained quantifiable below 15°C in Germany and Denmark (Fig. S3A and B).

**TABLE 2:** Spearman’s rank correlation coefficients (Bonferroni corrected) between the *Vibrio* species and *V. vulnificus* gene abundance, as quantified by ddPCR, in the water column and environmental and biological data^*a*^

Environmental and biological variables	Gene copies mL^−1^ *V. vulnificus* (Germany, *n* = 72)	Gene copies mL^−1^ *V*. *vulnificus* (*n* = 234)	Gene copies mL^−1^ total *Vibrio* spp. (*n* = 234)
	rho	rho	rho
Sea surface temperature (°C)	0.74^***^	0.27^***^	0.54^***^
Bacterial abundance (cells mL^−1^)	0.61^***^	0.21^***^	0.56^***^
Relative abundance of potential harmful cyanobacteria	0.59^***^	0.33^***^	0.21^***^
Gene copies *V. vulnificus* g^−1^ (sediment)	0.43^***^	0.23^***^	0.14^*^
NO_3_^−^ (µM)	0.32^**^	0.09	0.15^*^
Gene copies *Vibrio* spp. g^−1^ (sediment)	0.31^**^	0.05	0.51^***^
PO_4_^3−^ (µM)	0.28^**^	0.27^***^	0.21^***^
SSWWSH (m)	0.10	0.00	0.19^***^
NH_4_^+^ (µM)	0.03	−0.03	0.29^***^
Oxygen (mg L^−1^)	−0.19	0.18^**^	−0.21^***^
Salinity	−0.23	−0.30^***^	0.26^***^
Chlorophyll *a* (Chl *a*, mg m^−3^)	−0.29^**^	0.01	0.22^***^
Secchi depth (m)	−0.60^***^	−0.31^***^	−0.02

^*a*^ Asterisks indicate significant correlations (*P < 0.05, **P < 0.01, ***P < 0.001). The analysis includes 72 water samples for the German station and 234 water samples for all the stations. Note that SSWWSH is sea surface wind wave significant height.

### Environmental drivers and predictors of *Vibrio* spp. and *V. vulnificus*

**Fig 4:**
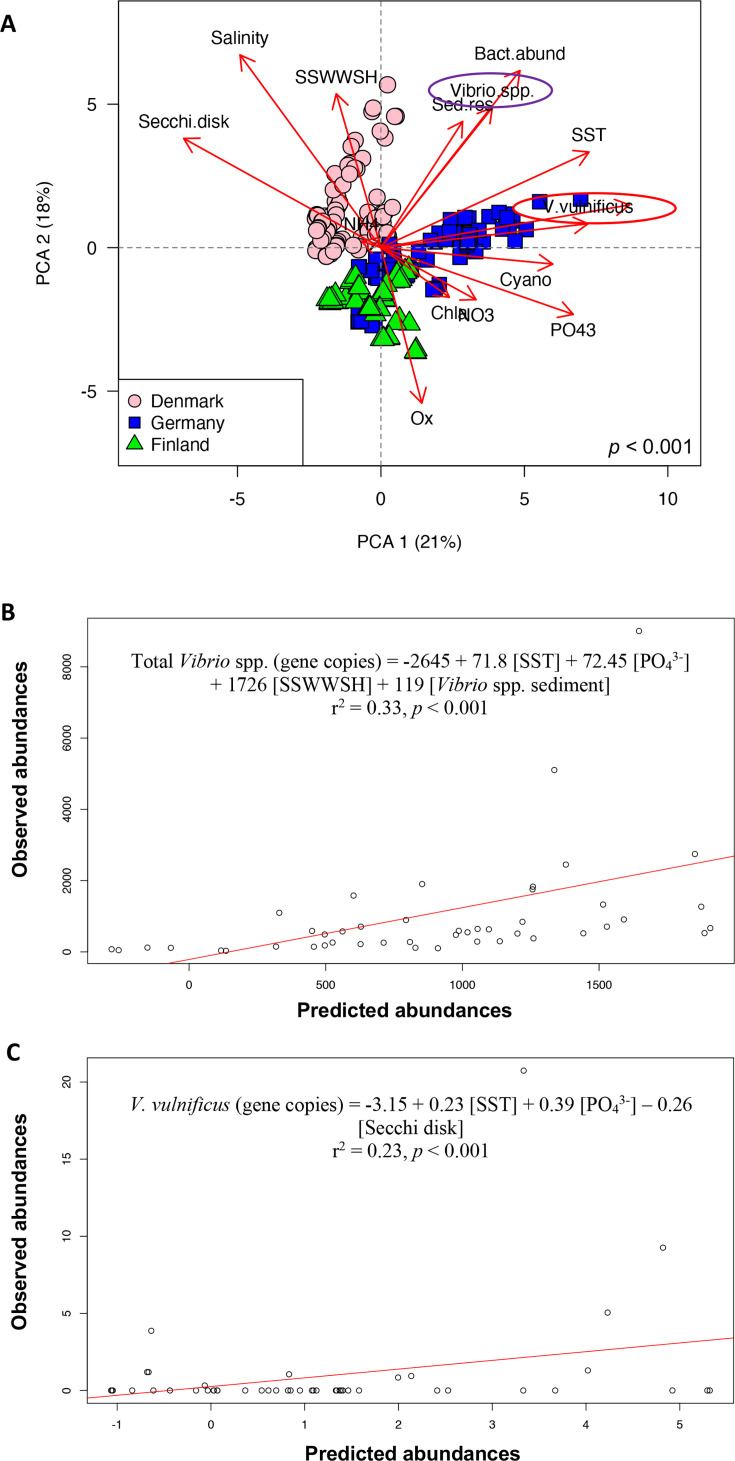
Environmental and biological drivers of *Vibrio* spp. and *V. vulnificus* occurrence in the water column. (**A**) PCA of the environmental and biological parameters measured from May to October (2022) in the Danish, Finnish, and German stations, including salinity, SST (°C), oxygen (Ox, mg L^−1^), chlorophyll *a* (Chl *a*, mg m^−3^), PO_4_^3−^ (µM), NH_4_^+^ (µM), NO_3_^−^ (µM), bacterial abundance (Bact.abund, cell mL^−1^), Secchi depth (m), SSWWSH (m), sediment resuspension (Sed.res, ASV from sediment detected in the water column), and relative abundance of potential harmful cyanobacteria, i.e., *Cyanobium*, *Dolichospermun*, and *Nodularia* (Cyano, %). Circles highlight *Vibrio* spp. and *V. vulnificus*. Observed vs predicted gene copies of (**B**) *Vibrio* spp. and (**C**) *V. vulnificus* from May to October (2022). Predictions were generated by stepwise multiple regression models, and the corresponding formula is shown. *Vibrio* species and *V. vulnificus* values in the PCA and model were based on the gene copies measured by ddPCR at the Danish, Finnish, and German stations. In (**A**), (**B**), and (**C**), all water samples were included, *n* = 234.

We investigated the environmental factors associated with the abundance of *Vibrio* spp. and *V. vulnificus*—gene copy number—within the water column across the sampled areas. A principal component analysis (PCA) revealed distinct environmental characteristics among the stations (Fig. 4A). Correlations between the variables analyzed are provided in Table S3. The abundances of *Vibrio* spp. and *V. vulnificus* were positively correlated with SST and negatively correlated with Secchi depth (Table 2; Fig. 4A). *Vibrio* spp. correlated positively with salinity, Chl *a*, PO_4_^3−^, NO_3_^−^, and NH_4_^+^ but negatively with oxygen (Table 2; Fig. 4A). *V. vulnificus* was positively correlated with PO_4_^3−^ and NO_3_^−^ (only at the German station), and restricted to a salinity of 8.1–11.2 (Table 2; Fig. 4A).

The environmental and biological factors that correlated with the gene copy numbers of *Vibrio* spp. and *V. vulnificus* were used to identify their key predictors. Our stepwise multiple regression model showed that SST, PO_4_^3–^, *Vibrio* species gene copy number from sediment, and sea surface wind wave significant height (SSWWSH) were key predictors of *Vibrio* spp. (Fig. 4B; r^2^ = 0.33, *P* < 0.001). For *V. vulnificus*, SST, PO_4_^3–^, and turbidity were the main predictors (Fig. 4C; r^2^ = 0.23, *P* < 0.001). The fact that *Vibrio* species gene copy number from sediment and turbidity appeared as key drivers of *Vibrio* species and *V. vulnificus* abundance, respectively, suggests a potential connection with sediment resuspension.

### The impact of sediment resuspension on *Vibrio* spp. and *V. vulnificus* in the water column

**Fig 5:**
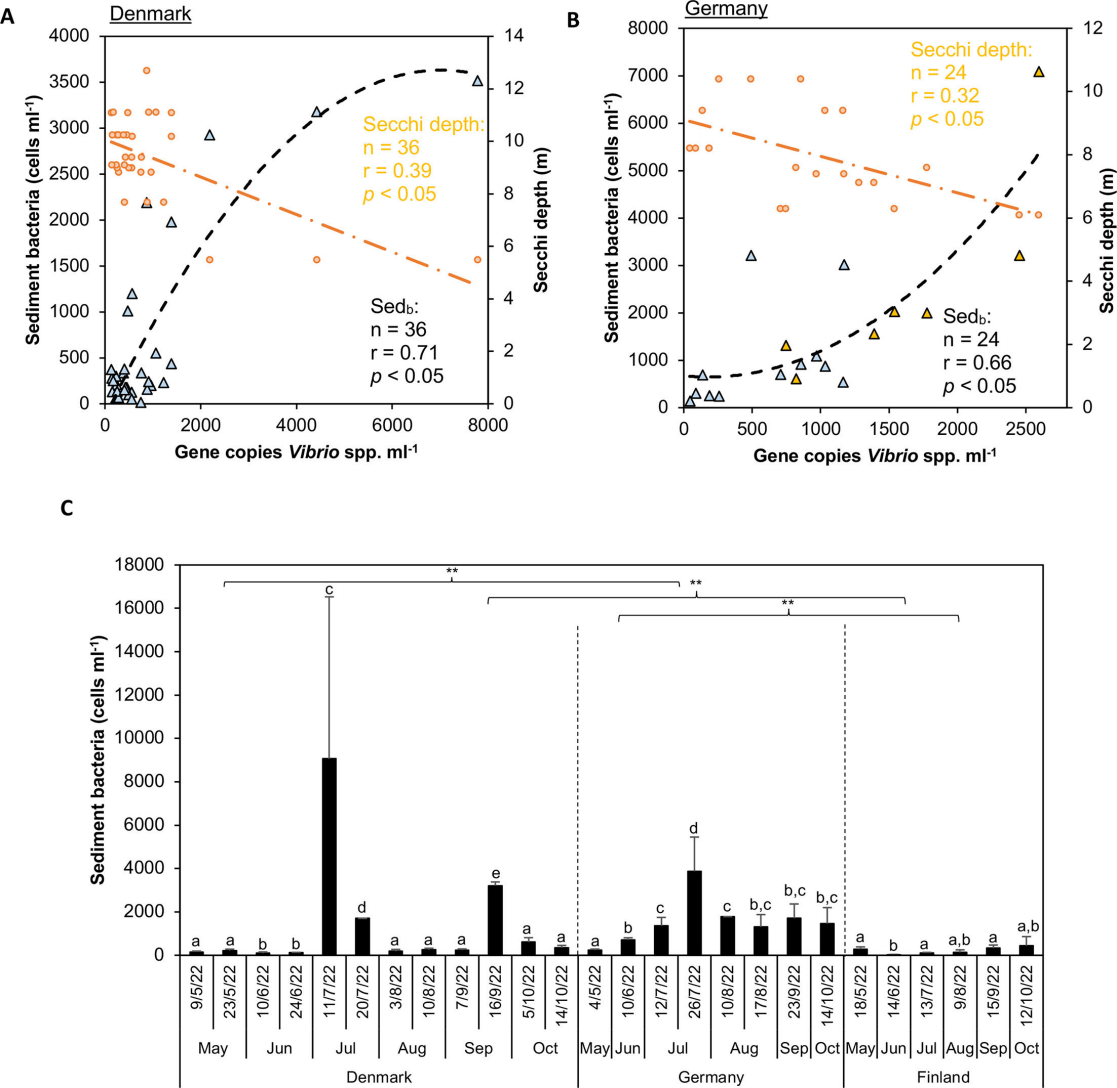
Regression scatterplots between sediment resuspension—proxied by sediment ASVs in the water column (their relative abundances multiplied by bacterial abundance [cells mL^−1^])—and the abundance of *Vibrio* spp. in the water column (triangles and black curves) (*P* < 0.05) in (**A**) Denmark and (**B**) Germany. In (**A**) and (**B**), a regression between the abundance of *Vibrio* spp. and Secchi depth (m), respectively, is inserted (dots and orange lines). In (**B**), the presence of *V. vulnificus* is indicated by orange triangles. Note that in (**A**) and (**B**), ddPCR values from the substations (A, B, and C) were averaged to enable comparisons with the pooled water samples that were sequenced. (**C**) Seasonality of sediment ASVs in the water column. Values are the average ± standard error (*n* = 3); letters show pairwise analysis among the variables (i.e., month) at each station; asterisks indicate pairwise significant differences between stations (*P < 0.05, **P < 0.01, ns [not significant] = *P* > 0.05), using a post hoc test (Wilcoxon) after a Kruskal–Wallis test.

To identify a potential link between sediment resuspension and elevated prevalence of *Vibrio* spp. in the water column, using DESeq2 (24), we identified ASVs that were characteristic of sediments and used their abundance (relative abundance multiplied by bacterial abundance) in the water column as an indication of sediment resuspension (Fig. S4A). These ASVs, which accounted for 3%–20% of sediment reads (<0.1% in the water column) and remained stable during the season, belonged mainly to the phyla *Actinobacteria*, *Chloroflexota*, *Desulfobacterota*, *Eisenbacteria*, *Gemmatimonadetes*, and *Myxococcota* (Fig. S4B and C). These phyla preferably inhabit the sediment (25–27). In Germany and Denmark, the *Vibrio* species gene copy number in water was positively correlated with the abundance of sediment bacteria, i.e., sediment resuspension, and negatively with Secchi depth (Fig. 5A and B). Interestingly, the presence of *V. vulnificus* in the water column in Germany coincided with markers of elevated sediment resuspension (Fig. 5B and C). Sediment resuspension was particularly high in July and September (Fig. 5C), and correlated with higher PO_4_^3–^ in the water column (Table S3). In Finland, there was no correlation between sediment resuspension and *Vibrio* spp., but fewer sediment bacteria were found in the water column compared to Denmark and Germany (Fig. 5C).

### Co-occurrence of specific microbial communities with *V. vulnificus*

**Fig 6:**
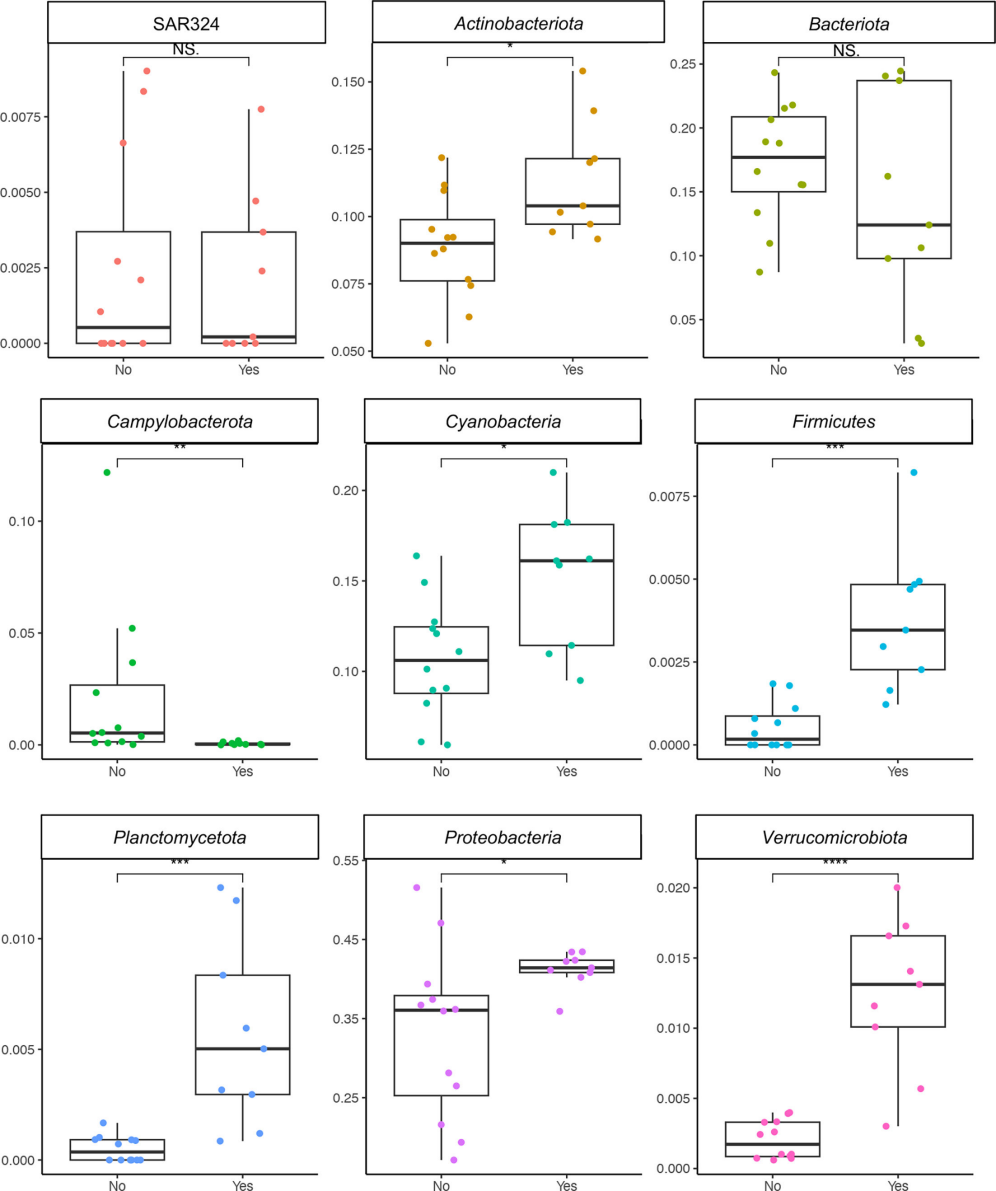
Significant changes in relative abundance of bacterial ASVs in the water column in the presence or absence of *V. vulnificus*. Approximately 900 ASVs showed notable shifts in their relative abundances when *V. vulnificus* was present at the German station. Phyla are shown. Values are the median, and vertical black lines indicate the position of the lower and upper quartiles. Asterisks indicate pairwise significant differences between the presence (“yes”) or absence (“no”) of *V. vulnificus* (*P < 0.05, **P < 0.01, NS [not significant] = *P* > 0.05), using a post hoc test (Wilcoxon) after a Kruskal–Wallis test.

As in our sediment resuspension analysis, we used DESeq2 to pinpoint the ASVs that displayed significance and dominance in the presence of *V. vulnificus*. At the German station, 900 ASVs in the water column significantly changed their relative abundance when *V. vulnificus* was present (Fig. S5A; top 30 most significant taxa *P* < 0.01). These belonged to *Actinobacteriota*, *Bacteriota*, *Campylobacterota*, *Firmicutes*, *Planctomycetota*, *Proteobacteria*, SAR324, and *Verrucomicrobiota* (Fig. 6). These phyla are known to exhibit a positive correlation with cyanobacterial and algae blooms (28). Indeed, ASVs within *Cyanobacteria* constituted up to 20% of the microbial community (Fig. 6, *P* < 0.05), correlating with periods of higher *Vibrio* species and *V. vulnificus* gene copy numbers (Fig. 5A and 6; Table 2). Hence, *Cyanobacteria* was one of the most abundant groups in the presence of *V. vulnificus* (Fig. 6; *P* < 0.01). The unicellular cyanobacteria *Cyanobium* spp., *Atelocyanobacterium thalassa* A (UCYN-A), and *Volcanococcus*, and the filamentous cyanobacteria *Dolichospermum* and *Nodularia* dominated the cyanobacterial community when *V. vulnificus* was present in the water (Fig. S5B). Note that the changes in cyanobacteria community structure were related to the temporal changes (Fig. S5B).

## DISCUSSION

The proliferation of *Vibrio* spp. in the Baltic Sea is a critical clinical challenge where the increased frequency of *Vibrio* infections appears to be closely linked to regional climatic trends and episodes of unusually warm weather (4, 29). Within the stations sampled, we found that *V. vulnificus* was almost exclusively confined to medium salinity (8.1–11.2), and episodes with elevated SST, high phosphate (PO_4_^3–^) concentration, and sediment resuspension served as indicators for *V. vulnificus* in the area. Our study did not show a reduction in *Vibrio* spp. associated with eelgrass beds, as suggested in an earlier Baltic Sea study (12), but rather indicates that vibriosis may be linked to sediment resuspension and its associated effects, e.g., elevated phosphate.

### Salinity is an important driver of *Vibrio* species communities

Low salinity caused a decrease in *Vibrio* species diversity but promoted specific *Vibrio* taxa. For instance, the fish pathogen *V. anguillarum*—known from aquaculture and larviculture industries in brackish waters (30)—dominated the *Vibrio* population in Finland and Germany (salinity 6.3–11.2). In contrast, at the more saline Danish station (salinity 13–21.3), the *Vibrio* species diversity was elevated, and the mussel pathogens *V. cyclitrophicus* and *V. chagasii* appeared during the summer months. In comparison, *V. vulnificus* was only present at the German station (salinity 8.1–11.2). Therefore, as previously suggested (7, 15, 31), salinity appears an important selective driver for *Vibrio* spp., but the proliferation of particular pathogens, e.g., *V. vulnificus*, is governed by the interplay of a range of environmental and biological factors (see below).

### The importance of temperature for *V. vulnificus* in the Baltic Sea

The analysis of the *Vibrio* community showed a strong seasonality, and SST was a key predictor for *Vibrio* spp. and *V. vulnificus* in the water column. This is consistent with previous observations of *Vibrio* spp. peaking during summer in other regions such as the Barnegat Bay, Chesapeake Bay, Monterrey Bay, Gulf of Mexico, North Sea, Atlantic Coast, and Sydney Harbour Estuary (15, 16, 19, 32–35). In contrast to *Vibrio* spp. that were found even below 10°C, *V. vulnificus* occurred in the water column only when temperatures were above 19°C, and peaked at 22°C. Interestingly, this aligns with a predicted increased probability of *V. vulnificus* in the 15–20°C range and a large risk of proliferation above 20°C (19). Hence, future longer heatwaves in the Baltic Sea region will likely increase the frequency of *V. vulnificus* during the summer season, as already reported in some recent studies (20–22), making *Vibrio* infections a putative increasing public health concern as this is the peak season for tourists and swimmers. However, unlike these earlier studies (20–22), *V. vulnificus* was almost exclusively found at the German station, at a medium salinity of 8.1–11.2. This observation carries significant implications as *V. vulnificus* is known to exist naturally across a broad spectrum of salinity levels, ranging from 5 to 25 (15, 36), with its ideal habitat falling within the 10 to 15 range (16). Hence, environmental drivers other than temperature and salinity regulate the prevalence of *V. vulnificus*.

### Role of phosphate for *V. vulnificus* in the Baltic Sea

PO_4_^3–^ was also a primary predictor for forecasting *V. vulnificus*. Consequently, regions such as the German station situated in the Mecklenburg Bight at the mouth of the Warnow River, known for substantial nutrient influx due to agricultural and industrial runoff (37, 38), are likely particularly prone to *V. vulnificus* outbreaks. These results deviate from previous findings suggesting that PO_4_^3–^ has a minimal explanatory effect on *Vibrio* species variance and exhibits no correlation with *V. vulnificus* (7), despite the earlier description of PO_4_^3–^ being an essential nutrient for *Vibrio* growth and pathogenesis (39, 40). However, this disparity could stem from the fact that most of these studies are conducted in Atlantic Ocean estuaries, where nutrient conditions may be different (31, 41, 42). Finally, we speculate that the association of *V. vulnificus* with high PO_4_^3–^ levels is indirect and *de facto* driven by sediment resuspension (discussed below).

Interestingly, we found a correlation between potentially harmful cyanobacteria and elevated levels of PO_3_^4–^ at the German station (Table S3), coinciding with a substantial increase in the gene copies of *V. vulnificus*. This is consistent with findings of our recent spatial study in the Baltic Sea (43). When *V. vulnificus* was present, the unicellular picocyanobacterium *Cyanobium* spp., commonly found in association with heterocystous bloom-forming cyanobacteria (44), and the filamentous and heterocystous cyanobacteria *Dolichospermum* and *Nodularia*, prevailed. They are key taxa in the harmful cyanobacterial blooms occurring every summer in the Baltic Sea Proper (45) and may release dissolved organic matter compounds specifically supporting pathogenic *Vibrio* spp. (46) , as well as providing surfaces for attachment, enhancing *V. vulnificus* survival (47). Collectively, the observed correlations between nutrient levels, cyanobacteria, and *V. vulnificus* emphasize the significance of PO_4_^3–^ concentration and rising temperatures as pivotal factors influencing the prevalence and survival of *V. vulnificus* in the Baltic Sea.

### Sediment as a source of *Vibrio* spp. in the water column

Our model showed that turbidity was one of the main drivers for *V. vulnificus* in the water column. Although we found specific cyanobacteria taxa co-occurring with *V. vulnificus*, our data showed a negative correlation between Chl *a* and gene copies of *V. vulnificus*, suggesting that turbidity could be mainly attributed to sediment resuspension rather than to phytoplankton biomass. This is supported by the positive correlation between sediment bacteria co-existing with *Vibrio* spp. and *V. vulnificus* in the water column, and the fact that some water samples contained *Vibrio* communities similar to those found in the sediment, suggesting a benthic-pelagic coupling. The detection of these sediment bacteria by sequencing in the water column can be a more useful proxy for sediment resuspension than turbidity, e.g., estimated by Secchi depth, which can be highly influenced by organic matter load or phytoplankton blooms (48). Our findings are consistent with other marine studies connecting *Vibrio* spp. and sediment resuspension, whether due to human activity, e.g., ship traffic, or natural phenomena like rainfalls and storms (31, 49, 50).

We speculate that sediment resuspension can increase *V. vulnificus* levels in the water column through two related mechanisms. First, by introducing *V. vulnificus* found in association with particulate matter originating from the sediment. Sediments may harbor pathogens (51, 52), and sediments rich in silt and organic matter—as those found in Germany—may harbor a particularly high density of bacterial pathogens (51), which may be released to the water column (31). Indeed, gene copies of *Vibrio* spp. and *V. vulnificus* were 100 and 1,000 times higher in the sediment than in the water, respectively, indicating that *Vibrio* populations persist and thrive in sediment (16). Our data show that *V. vulnificus* remained quantifiable in sediments below 15°C, indicating that sediment can be a *V. vulnificus* reservoir even when the water is less warm. At these temperatures, *Vibrio* spp., including *V. vulnificus*, can enter a viable but non-culturable (VBNC) latency state, enabling survival even at low temperatures (53). However, the proportion of bacteria capable of entering this state cannot be accurately assessed using the methods employed in this study. Second, by supplying PO_4_^3–^ but also organic matter to the water column, thereby promoting the growth of *V. vulnificus*. We showed that sediment resuspension events correlated with higher PO_4_^3–^ into the water column. It is known that sediments can serve as a source of PO_4_^3–^, and their release may depend on their composition, which differed between stations (Table S3), and oxygen conditions (54). The accumulation of PO_4_^3–^ in sediment can be more easily released into the water column during summer, as depicted in Figure 1E. This phenomenon can be attributed to the heightened temperatures, which augment solubility, sediment adsorption capacity, and organic matter dynamics (55), leading to changes in nutrient availability and ecological processes indirectly impacting the prevalence of this pathogenic bacterium (56). This scenario may be occurring at the German station, where the sampling location and sediment composition could be influencing these effects. From our study, sediment resuspension appears a key driver of pathogenic *Vibrio* spp. in the water column; however, it is not possible to disentangle the relative importance of the two scenarios, i.e., distinguishing between cell effects, organic matter, and PO_4_^3–^ transfer associated with resuspension.

### Conclusions

Our study identified SST, PO_4_^3–^, and sediment resuspension as key factors controlling the occurrence of pathogenic *Vibrio* spp., such as *V. vulnificus*, in the Baltic Sea. In addition, *V. vulnificus* was only present at intermediate salinity. Climate change will cause an increased frequency of natural disasters and rain-driven floods in the Baltic Sea area with associated increased sediment resuspension and elevated nutrient concentrations (57), likely leading to raised levels of vibriosis, as was observed in association with hurricanes in the USA (58). Although it is conceivable that seagrass meadows may indirectly prevent *Vibrio* species proliferation via effects on sedimentation, sediment surface stabilization, and mitigation of turbulent flow (12), we did not find a reduction in *Vibrio* spp. associated with eelgrass beds. Hereby, our study contradicts the assumption that seagrass reduce pathogenic *Vibrio* species levels in the areas studied. This is also supported by our recent spatial study, in which, as in the current study, we employed culture-independent methods and found that *V. vulnificus* abundance did not vary significantly between vegetated and non-vegetated areas in the Baltic Sea (43). Accordingly, seagrass meadows do not appear an effective nature-based solution for reducing *V. vulnificus* abundance and associated infections in the Baltic Sea, and alternative solutions to mitigate the vibriosis problem should be explored.

## MATERIALS AND METHODS

### Sampling

Three coastal stations along the southwest-northeast salinity gradient of the Baltic Sea were sampled from May to October 2022 (Table 1; Fig. 1A): Julebæk, in the Øresund Strait (Denmark), located in the Hovedstaden region, an area impacted by extensive shipping and high winds and currents; Warnemünde, at the mouth of the Warnow River (Germany), a touristic area in the Mecklenburg-Vorpommern region; and Ängsö Bay, situated in the Archipelago Sea (Southwest Finland), located to the west of Ängsö Island, in an area characterized by a low population density (Table 1). Samplings were carried out once or twice per month by snorkeling or diving (scuba) with local boats at three sampling points per station, i.e., substation: A, in the middle; B, 15 m; and C, 100 m outside of the *Z. marina* meadow. Based on data collected in summer 2021 (43), the average abundance and length of the seagrass beds sampled in Denmark, Germany, and Finland were 675 shoots m^−2^ and 74.26 cm, 400 shoots m^−2^ and 75.55 cm, and 225 shoots m^−2^ and 22.46 cm, respectively. When sampling, food colorant was employed to monitor the current, and divers navigated toward the sampling site against the current while carefully controlling buoyancy to avoid sediment resuspension. First, water samples were collected by gentle suction of 1.5-L water using three acid-washed 500-mL syringes per replicate. The syringes were rinsed three times with sample water prior to use. The water was used for the downstream analysis, i.e., DNA, chlorophylls, nutrient, and flow cytometry. Water samples were collected either 5 cm from the eelgrass leaves (to closely monitor the maximum effect of the eelgrass) at the substation A or 20 cm above the sediment level to avoid any kind of sediment resuspension at the substations B and C. Second, eelgrass leaves (only in A) and the uppermost sediment layer were collected in triplicate 50-mL Falcon tubes. All samples were stored in a cooler and processed within 2 h. SST, salinity, and oxygen were measured at each sampling. Secchi depth and SSWWSH values were obtained from Copernicus EMS using the three station coordinates and the date and time at which sampling was conducted (Table S1).

### Sample processing

Water samples (500 mL) for DNA and chlorophyll *a* (Chl *a*) analyses were immediately filtered onto a Durapore membrane filter (0.22 µm, Sigma-Aldrich, Massachusetts, USA) and GF/F filter (Whatman, Sigma-Aldrich, Massachusetts, USA), respectively, and stored at −20°C. Filtered water was stored frozen for analysis of phosphate (PO_4_^3–^), nitrate (NO_3_^–^), and ammonium (NH_4_^+^). For bacterial enumeration, 2 mL of water was fixed with formaldehyde 37% (vol/vol), incubated for 1 h at 8°C, shock frozen, and stored at −80°C. Sediment and eelgrass samples were immediately frozen at −20°C.

### Chlorophyll *a,* inorganic nutrients, bacterial abundance, and grain size

Chl *a* was extracted with 96% (vol/vol) ethanol and was measured on a Trilogy Laboratory Fluorometer (Turner Designs, San Jose, CA, USA) calibrated with a Chl *a* standard (DHI, Denmark) following Jespersen and Christoffersen (59). NH_4_^+^ was quantified fluorometrically, according to Holmes et al. (60), whereas PO_4_^−3^ and NO_3_^−^ were quantified using standard colorimetric methods (61, 62). Bacteria were enumerated on a FACSCanto II flow cytometer (BD, New Jersey, USA) according to Brussaard et al. (63) using TrueCount beads (BD) to measure the flow rate. Grain size from 27 sediment samples between July and September (*n* = 9 per station) was measured on the Mastersizer 3000 (Malvern Panalytical, Malvern, UK).

### Molecular analyses

#### DNA extraction

Durapore membrane filters were thawed and ground in 2-mL Eppendorf tubes with liquid nitrogen. The sediment or eelgrass samples were washed before extraction with 1.5 mL of phosphate buffer saline (PBS) in a 2-mL tube containing approximately 1 g of sediment or 0.3 g of eelgrass to minimize contamination from the water column. DNA was extracted using the DNeasy PowerSoil Pro Kit (Qiagen, Hilden, Germany) following the manufacturer’s instructions. Samples were eluted in 10 mM Tris buffer (pH 8), and DNA concentration was quantified using the PicoGreen dsDNA Assay Kit (Thermo Fisher, Massachusetts, USA).

#### 16S rRNA amplicon sequencing analysis and *Vibrio* species annotation

DNA from triplicate water samples from each substation was combined at every sampling point, i.e., uniformly mixed, resulting in one water sample sequenced per substation. Sediment and eelgrass samples were sequenced in triplicates. Thus, DNA from 78 water, 204 sediment, and 78 eelgrass samples was sequenced at the SciLifeLab National Genomics Infrastructure (NGI, Solna, Sweden). Libraries were prepared using the primers 341F/805R (341F, 5′-CCTACGGGNGGCWGCAG-3′; 805R, 5′-GACTACHVGGGTATCTAATCC-3′) targeting the hypervariable V3-V4 region of the 16S rRNA gene (64). Library preparation was performed according to the NGI protocols (https://ngisweden.scilifelab.se/methods/illumina-16s-sequencing-2/ and https://ngisweden.scilifelab.se/2021/01/tech-note-increase-complexity-of-amplicon-libraries-using-phased-primers/). Dual-index libraries were created according to the NGI protocols, and that 301 bp paired-end sequencing was performed on a NextSeq2000 instrument with a “P1” flowcell. After sequencing, the library was demultiplexed, and phased primers, Illumina adaptors, and 16S rRNA gene primers were removed (https://github.com/biodiversitydata-se/amplicon-multi-cutadapt). Sequence analysis was performed in R Studio v 4.1.0. (http://www.r-project.org). Three of the 78 water samples did not pass the quality control. ASVs were generated with the DADA2 pipeline v.1.21.0 (59) and annotated using a curated version of 16S rRNA genes from Genome Taxonomy Database (GTDB) (v.R06-RS202-1) (60). Sequences were deposited in NCBI (PRJNA1011541).

The taxonomic annotation of the 16S ASVs was carried out in two steps. First, we used the DADA2 function assignTaxonomy (65) and 16S sequences from GTDB to get taxonomic annotations of the ASVs to genus level (66). Second, for the ASVs annotated as *Vibrio* in the initial annotation, species-level classification was achieved by sequence comparison to a custom database (BLAST, V. 2.13.0) (67). This database includes 16S rRNA gene sequences of complete *Vibrio* genomes from RefSeq (51 species, 317 strains—including 22 *V*. *vulnificus* strains) (68), 41 draft *V. vulnificus* genomes from clinical isolates from the Baltic region (4, 6), and 84 draft *V. vulnificus* genomes from environmental Baltic Sea isolates (Delgado et al., unpublished data). For an ASV to obtain a species-level assignment, we required perfect (100% identity) alignment of the full ASV to 16S rRNA gene(s) of a single species in the custom database. In some cases, this resulted in ambiguous species annotation. However, when considering matches for *V. vulnificus*, the associations were unambiguous, meaning that the identified ASVs perfectly aligned with 16S sequences belonging exclusively to this species.

Exact match is recommended for species-level assignments of 16S ASVs (69). The scripts used for the bioinformatics processing are available at https://github.com/lfdelzam/ASV_dada2_chunck/ and the 16S rRNA *Vibrio* database for this paper is available at https://zenodo.org/records/1087.

#### Quantification of *Vibrio* spp. and *V. vulnificus* with digital droplet PCR (ddPCR)

The abundance of *Vibrio* spp. and *V. vulnificus* in water and sediments was assessed by gene quantification using ddPCR. We targeted the 16S rRNA gene with 567F/680R (567F, 5′-GGCGTAAAGCGCATGCAGGT-3′; 680R, 5′-GAAATTCTACCCCCCTCTACAG-3′ [32]) for total *Vibrio* spp., and *vvhA* gene with vvh-785f/990r (vvh-785f: 5′-TTCCAACTTCAAACCGAACTATGAC-3′, vvh-990r: 5′-ATTCCAGTCGATGCGAATACGTTG-3′ [70]) for *V. vulnificus*. For the ddPCR reaction, 11-µL QX200 ddPCR EvaGreen Supermix (Bio-Rad, München, Germany), 0.01 µM forward and reverse primer (final concentration), 5–15 ng DNA template, and diethylpyrocarbonate (DEPC) H_2_O were added in a final volume of 22 µL. Droplets were generated using a QX100 droplet generator (Bio-Rad, München, Germany) according to the manufacturer’s instructions. The emulsified samples were transferred to a 96-well plate and sealed with a pierceable heat-sealing film (Bio-Rad, München, Germany). PCR was performed using a Bio-Rad C1000 Touch thermal cycler (Bio-Rad, München, Germany). The PCR conditions for *Vibrio* spp. were 94°C for 5 min, 40 cycles of 94°C for 1 min, 61.6°C for 1 min, and 72°C for 1 min. For *V. vulnificus*, the program was 95°C for 5 min, followed by 40 cycles at 95°C for 30 s, 58.5°C for 1 min, and 72°C for 1 min. In both cases, a final step of 4°C for 5 min, 90°C for 5 min, and an infinite hold at 4°C was performed. The plate was then analyzed with the QX200 Droplet Digital PCR system (Bio-Rad) using Quantasoft 1.74.09.17 software (Bio-Rad, 181–4040, München, Germany). Positive controls of *V. vulnificus* isolates and blanks with DEPC water were used. Based on the above described 16S rRNA gene sequencing, we applied a correction factor for *Vibrio* species counts, i.e., the ratio of *Vibrio* spp. and *Photobacterium* spp. 16S rRNA gene sequences, to account for the cross-reaction of the primer set 567F/680R with *Photobacterium* spp., *sensu* (71).

### Community analysis, statistics, and modeling

All analyses were carried out using R Studio v.4.1.0.

#### Community analysis

Sample data matrices were managed using the phyloseq package v.1.36 (72). Community analyses were exclusively carried out on ASVs within the *Vibrio* genus unless stated otherwise. Alpha diversity (Shannon index) was calculated after rarefaction. PCA and redundancy analysis (RDA) were performed with the microViz package v.0.10.8 (72) to measure the similarity or dissimilarity between *Vibrio* communities using central log ratio (clr)-transformed data.

To evaluate changes in the *Vibrio* community, we conducted a permutational multivariate analysis of variance (PERMANOVA) using distance matrices (number of permutations = 999) using the Euclidean distance matrix. We also used an analysis of variance (ANOVA)-like permutation test for constrained correspondence analysis to determine the significance of the constraints (number of permutations = 999) using the anova.cca, which is part of vegan v.2.6.4 (73). To evaluate pairwise differences between groups, pairwise analyses were carried out with PairwiseAdonis v.0.4 (74). Additionally, we performed a similarity percentage (SIMPER) analysis (number of permutations = 999) to identify the taxa responsible for differences between groups of samples, using vegan v.2.6.4 (73).

To identify ASVs that were differentially abundant in the sediment, a negative binomial Wald test was conducted using DESeq2 v.1.32.0 on the non-rarified and non-clr-transformed data (24). The sediment samples (*n* = 204) were compared to the respective water samples (*n* = 75). Despite unequal sample sizes, the data exhibited a negative binomial distribution, ensuring a robust analysis. Significance was set at *P* < 0.01. The top 26 most significant ASVs differentially abundant in the sediment at each station were used as indicators for sediment resuspension when found in the water column. Additionally, the same analysis was performed to determine the ASVs associated with the presence (*n* = 9) and absence (*n* = 12) of *V. vulnificus* (based on the ddPCR values). DESeq2 analysis was carried out with the top 10,000 most abundant ASVs (comprising almost 100% of the entire microbial community). The ASVs detected were visualized using clustering heatmaps created with the complexHeatmap package v.2.8.0 (75).

#### Statistical analyses

Homogeneity of variances for the ddPCR and relative abundance data followed a non-normal distribution (Leven’s test). Therefore, non-parametric analyses were employed using Kruskal–Wallis. Pairwise comparisons were performed using Wilcoxon rank sum tests. Spearman’s rank analysis was employed to determine relationships between the ddPCR data of *Vibrio* spp. and *V. vulnificus* in the water column and environmental and biological data (*n* = 234), using Bonferroni correction to adjust Spearman rank correlation *P* values. Unless otherwise stated, the significance level for all analyses, including correlations, was set to *P* < 0.05.

#### Prediction of *Vibrio* spp. and *V. vulnificus*

To predict *Vibrio* species and *V. vulnificus* occurrence, we used stepwise multiple regressions, following the methodology outlined previously (33, 76). The data used for training and testing the model were split, with 80% used for training and 20% for testing. The analysis was performed using the olsrr package v0.5.3 (https://CRAN.R-project.org/package=olsrr), and the predictive model was developed based on the ddPCR data obtained from the water column (*n* = 234). We tested that the data met the assumptions of linearity, independence, normality of residuals, and homoscedasticity. Variables exhibiting high co-linearity were eliminated. Only variables with *P* < 0.05 were included in the model. Studentized residual analyses were conducted to thoroughly examine the variance between observed and predicted abundances. The visual examination of the difference between predicted and observed abundances was calculated and plotted.

## Data Availability

The 16S rRNA sequences are available in NCBI (PRJNA1011541), and the scripts used for the bioinformatics processing are accessible at https://github.com/lfdelzam/ASV_dada2_chunck/. The 16S rRNA *Vibrio* database for this paper is available at https://zenodo.org/records/1087.

## SUPPLEMENTAL MATERIAL

Supplemental material mbio.01569-24-s0001.pdf Tables S1–S3; Fig. S1–S5.

ASM does not own the copyrights to Supplemental Material that may be linked to, or accessed through, an article. The authors have granted ASM a non-exclusive, world-wide license to publish the Supplemental Material files. Please contact the corresponding author directly for reuse.
