# Supplementary material for: Temperature, sediment resuspension, and salinity drive the prevalence of Vibrio vulnificus in the coastal Baltic Sea
Source: mBio. 2024 Sep 19;15(10):e01569-24. doi: 10.1128/mbio.01569-24 (PMC11481517; doi:10.1128/mbio.01569-24)
Supplement: Supplemental material — Tables S1–S3; Fig. S1–S5. [file mbio.01569-24-s0001.pdf]

Supplementary materials

Temperature, sediment resuspension, and salinity drive the prevalence of *Vibrio vulnificus* in the coastal Baltic Sea

Víctor Fernández-Juárez[1]*, David J. Riedinger[2], Joao Bosco Gusmao[3], Luis Fernando Delgado-Zambrano[4], Guillem Coll-García[5,6], Vasiliki Papazachariou[1], Daniel P.R. Herleman[7], Christian Pansch[3], Anders F. Andersson[4], Matthias Labrenz[2], Lasse Riemann[1]*

[1]Marine Biological Section, Department of Biology, University of Copenhagen, Copenhagen, Denmark

[2]Department of Biological Oceanography, Leibniz Institute for Baltic Sea Research Warnemünde (IOW), Rostock, Germany

[3]Environmental and Marine Biology, Åbo Akademi University, Turku, Finland

[4]Science for Life Laboratory, Department of Gene Technology, KTH Royal Institute of Technology, 171 21, Stockholm, Sweden

[5]Microbiology, Biology Department, University of the Balearic Islands, Palma de Mallorca, Spain

[6]Environmental Microbiology Group, Mediterranean Institute for Advanced Studies (CSIC-UIB), Esporles, Spain

[7]Estonian University of Life Sciences, Fr. R. Kreutzwaldi 1, Tartu, Estonia

*Corresponding authors: Víctor Fernández-Juárez (email: victor.fj@bio.ku.dk) and Lasse Riemann (lriemann@bio.ku.dk)

Table S1. Metadata for water samples collected from May to October 2022. The values presented are the averages obtained from triplicate samples. SST: sea surface temperature.

Station_ID	Substation	Sample type	Latitute	Longitude	Month	Date	Country	Salinity	SST (°C)	NH_4^+ (µM)	Heterotrophic cells mL^{-1}	NO_3^- (µM)	PO_4^{3-} (µM)
BV01-1	A	water	56.058401	12.577956	May	09/05/2022	Denmark	12.7	11.8	0.3	4.12E+05	0.09	0.06
BV01-1	B	water	56.058692	12.578673	May	09/05/2022	Denmark	12.7	11.8	0.2	3.98E+05	0.12	0.08
BV01-1	C	water	56.058401	12.580089	May	09/05/2022	Denmark	12.8	11.8	0.2	3.78E+05	0.12	0.08
BV01-2	A	water	56.058401	12.577956	May	23/05/2022	Denmark	14.6	13.1	0.8	4.76E+05	0.08	0.06
BV01-2	B	water	56.058692	12.578673	May	23/05/2022	Denmark	14.6	13.2	0.3	6.23E+05	0.10	0.06
BV01-2	C	water	56.058401	12.580089	May	23/05/2022	Denmark	15.2	13.2	0.3	5.77E+05	0.10	0.06
BV01-3	A	water	56.058401	12.577956	June	10/06/2022	Denmark	15.3	14.6	0.5	5.49E+05	0.11	0.07
BV01-3	B	water	56.058692	12.578673	June	10/06/2022	Denmark	15.3	14.6	0.1	5.47E+05	0.09	0.07
BV01-3	C	water	56.058401	12.580089	June	10/06/2022	Denmark	15.2	14.5	0.1	5.64E+05	0.09	0.07
BV01-4	A	water	56.058401	12.577956	June	24/06/2022	Denmark	16.7	17.8	0.2	1.01E+06	0.11	0.06
BV01-4	B	water	56.058692	12.578673	June	24/06/2022	Denmark	16.7	17.8	0.5	9.19E+05	0.11	0.07
BV01-4	C	water	56.058401	12.580089	June	24/06/2022	Denmark	16.7	17.2	1.2	5.28E+05	0.11	0.07
BV01-5	A	water	56.058401	12.577956	July	11/07/2022	Denmark	16.6	17.9	1.4	2.57E+06	0.10	0.06
BV01-5	B	water	56.058692	12.578673	July	11/07/2022	Denmark	16.6	17.9	0.3	2.53E+06	0.08	0.08
BV01-5	C	water	56.058401	12.580089	July	11/07/2022	Denmark	16.6	17.9	0.1	2.13E+06	0.08	0.08
BV01-6	A	water	56.058401	12.577956	July	20/07/2022	Denmark	17.5	19.8	0.1	1.20E+06	0.11	0.06
BV01-6	B	water	56.058692	12.578673	July	20/07/2022	Denmark	13.1	19.5	0.2	1.19E+06	0.10	0.07
BV01-6	C	water	56.058401	12.580089	July	20/07/2022	Denmark	13.1	19.5	0.2	1.04E+06	0.10	0.07
BV01-7	A	water	56.058401	12.577956	August	03/08/2022	Denmark	14.9	18.0	0.3	8.82E+05	0.10	0.07
BV01-7	B	water	56.058692	12.578673	August	03/08/2022	Denmark	14.9	18.0	0.1	1.34E+06	0.11	0.08
BV01-7	C	water	56.058401	12.580089	August	03/08/2022	Denmark	14.9	18.0	0.1	7.34E+05	0.11	0.08
BV01-8	A	water	56.058401	12.577956	August	10/08/2022	Denmark	14.4	18.1	0.2	1.19E+06	0.11	0.07
BV01-8	B	water	56.058692	12.578673	August	10/08/2022	Denmark	14.4	18.1	0.1	1.46E+06	0.12	0.07
BV01-8	C	water	56.058401	12.580089	August	10/08/2022	Denmark	14.4	18.1	0.2	9.43E+05	0.12	0.07
BV01-9	A	water	56.058401	12.577956	September	07/09/2022	Denmark	14.9	16.6	0.2	1.62E+06	0.08	0.05
BV01-9	B	water	56.058692	12.578673	September	07/09/2022	Denmark	14.9	16.6	0.5	1.20E+06	0.10	0.05

BV01-9	C	water	56.058401	12.580089	September	07/09/2022	Denmark	13.9	17.3	0.7	1.32E+06	0.10	0.05
BV01-10	A	water	56.058401	12.577956	September	16/09/2022	Denmark	16.6	15.6	0.2	1.80E+06	0.20	0.06
BV01-10	B	water	56.058692	12.578673	September	16/09/2022	Denmark	16.6	15.6	0.1	1.31E+06	0.10	0.07
BV01-10	C	water	56.058401	12.580089	September	16/09/2022	Denmark	18.8	16.3	0.2	1.03E+06	0.10	0.07
BV01-11	A	water	56.058401	12.577956	October	05/10/2022	Denmark	16.6	13.6	0.2	8.44E+05	0.10	0.06
BV01-11	B	water	56.058692	12.578673	October	05/10/2022	Denmark	16.6	13.6	0.1	1.24E+06	0.10	0.06
BV01-11	C	water	56.058401	12.580089	October	05/10/2022	Denmark	16.6	13.6	0.1	1.11E+06	0.10	0.06
BV01-12	A	water	56.058401	12.577956	October	14/10/2022	Denmark	21.3	12.6	0.1	4.58E+05	0.12	0.07
BV01-12	B	water	56.058692	12.578673	October	14/10/2022	Denmark	21.3	12.6	0.1	4.62E+05	0.12	0.09
BV01-12	C	water	56.058401	12.580089	October	14/10/2022	Denmark	21.3	12.6	0.0	4.63E+05	0.12	0.09
BV02-1	A	water	54.179579	12.103401	May	04/05/2022	Germany	8.7	11.8	0.1	3.89E+05	0.12	0.08
BV02-1	B	water	54.179639	12.103105	May	04/05/2022	Germany	8.7	12.5	0.5	3.33E+05	0.09	0.05
BV02-1	C	water	54.1788	12.106677	May	04/05/2022	Germany	8.7	13.2	0.1	4.47E+05	0.09	0.05
BV02-2	A	water	54.179579	12.103401	June	10/06/2022	Germany	10.4	17.4	0.0	4.20E+05	0.09	0.07
BV02-2	B	water	54.179639	12.103105	June	10/06/2022	Germany	10.7	17.7	0.2	6.70E+05	0.08	0.09
BV02-2	C	water	54.1788	12.106677	June	10/06/2022	Germany	10.7	18.4	0.2	8.03E+05	0.08	0.09
BV02-3	A	water	54.179579	12.103401	July	12/07/2022	Germany	8.3	18.9	0.1	1.45E+06	0.10	0.10
BV02-3	B	water	54.179639	12.103105	July	12/07/2022	Germany	8.2	19.4	0.2	1.60E+06	0.09	0.12
BV02-3	C	water	54.1788	12.106677	July	12/07/2022	Germany	8.3	19.2	0.2	1.43E+06	0.09	0.12
BV02-4	A	water	54.179579	12.103401	July	26/07/2022	Germany	9.9	19.5	0.4	1.22E+06	0.14	0.12
BV02-4	B	water	54.179639	12.103105	July	26/07/2022	Germany	10.0	19.5	0.4	1.83E+06	0.22	0.15
BV02-4	C	water	54.1788	12.106677	July	26/07/2022	Germany	9.9	19.4	0.4	1.94E+06	0.22	0.15
BV02-5	A	water	54.179579	12.103401	August	10/08/2022	Germany	9.5	21.5	0.1	1.27E+06	0.12	0.10
BV02-5	B	water	54.179639	12.103105	August	10/08/2022	Germany	9.7	21.3	0.2	9.70E+05	0.11	0.12
BV02-5	C	water	54.1788	12.106677	August	10/08/2022	Germany	9.8	21.5	0.1	2.32E+06	0.11	0.12
BV02-6	A	water	54.179579	12.103401	August	17/08/2022	Germany	10.1	21.6	0.2	3.29E+05	0.11	0.08
BV02-6	B	water	54.179639	12.103105	August	17/08/2022	Germany	9.8	21.9	0.2	7.96E+05	0.11	0.09
BV02-6	C	water	54.1788	12.106677	August	17/08/2022	Germany	9.2	20.5	0.1	1.27E+06	0.11	0.09
BV02-7	A	water	54.179579	12.103401	September	23/09/2022	Germany	10.2	15.2	0.1	6.17E+05	0.17	0.18

BV02-7	B	water	54.179639	12.103105	September	23/09/2022	Germany	10.2	15.4	0.0	7.20E+05	0.10	0.14
BV02-7	C	water	54.1788	12.106677	September	23/09/2022	Germany	11.0	15.8	0.2	4.90E+05	0.10	0.14
BV02-8	A	water	54.179579	12.103401	October	14/10/2022	Germany	10.8	13.4	0.4	6.83E+05	0.17	0.14
BV02-8	B	water	54.179639	12.103105	October	14/10/2022	Germany	11.4	13.5	0.5	7.51E+05	0.13	0.12
BV02-8	C	water	54.1788	12.106677	October	14/10/2022	Germany	11.2	13.6	0.0	6.99E+05	0.13	0.12
BV03-1	A	water	60.1087	21.7119	May	18/05/2022	Finland	6.5	7.0	0.2	3.96E+05	0.10	0.08
BV03-1	B	water	60.1087	21.7119	May	18/05/2022	Finland	6.5	7.0	0.1	5.16E+05	0.09	0.08
BV03-1	C	water	60.1087	21.7119	May	18/05/2022	Finland	6.5	7.0	0.1	5.49E+05	0.09	0.08
BV03-2	A	water	60.1087	21.7119	June	14/06/2022	Finland	6.4	13.5	0.1	6.59E+05	0.09	0.09
BV03-2	B	water	60.1087	21.7119	June	14/06/2022	Finland	6.4	14.4	0.1	8.87E+05	0.09	0.05
BV03-2	C	water	60.1087	21.7119	June	14/06/2022	Finland	6.4	14.5	0.0	8.46E+05	0.09	0.05
BV03-3	A	water	60.1087	21.7119	July	13/07/2022	Finland	6.5	19.1	0.1	9.06E+05	0.10	0.08
BV03-3	B	water	60.1087	21.7119	July	13/07/2022	Finland	6.5	19.2	0.1	6.05E+05	0.08	0.07
BV03-3	C	water	60.1087	21.7119	July	13/07/2022	Finland	6.5	19.5	0.1	1.19E+06	0.08	0.07
BV03-4	A	water	60.1087	21.7119	August	09/08/2022	Finland	6.3	18.5	0.0	3.73E+05	0.10	0.08
BV03-4	B	water	60.1087	21.7119	August	09/08/2022	Finland	6.3	18.3	0.4	4.95E+05	0.08	0.06
BV03-4	C	water	60.1087	21.7119	August	09/08/2022	Finland	6.3	19.1	0.0	4.41E+05	0.08	0.06
BV03-5	A	water	60.1087	21.7119	September	15/09/2022	Finland	6.4	15.7	0.1	3.31E+05	0.13	0.09
BV03-5	B	water	60.1087	21.7119	September	15/09/2022	Finland	6.4	15.3	0.0	2.94E+05	0.09	0.09
BV03-5	C	water	60.1087	21.7119	September	15/09/2022	Finland	6.4	15.7	0.0	2.83E+05	0.09	0.09
BV03-6	A	water	60.1087	21.7119	October	12/10/2022	Finland	6.4	12.2	0.2	3.24E+05	0.40	0.17
BV03-6	B	water	60.1087	21.7119	October	12/10/2022	Finland	6.4	12.0	0.1	3.52E+05	0.23	0.14
BV03-6	C	water	60.1087	21.7119	October	12/10/2022	Finland	6.4	11.9	0.2	2.90E+05	0.23	0.14

Table S2. Grain size composition. For the analysis, 27 sediment samples obtained between July and September (n = 9 per station) were selected from the three stations. The values are the average ± standard error.

	**Grain size composition %**				
	**Clay**	**Silt**	**Sand fine**	**Sand medium**	**Sand coarse**
**Denmark**	0.00 ± 0.00	4.02 ± 3.35	87.49 ± 16.95	8.49 ± 3.84	0.00 ± 0.00
**Germany**	0.02 ± 0.00	6.55 ± 1.62	79.64 ± 13.89	13.80 ± 3.42	0.03 ± 0.00
**Finland**	0.00 ± 0.00	1.16 ± 1.93	53.53 ± 12.28	45.09 ± 3.00	0.77 ± 0.00

Table S3. Spearman's rank correlation coefficients (Bonferroni corrected) between environmental parameters measured in the water column at the three stations. Colors indicate significant correlations (green, $p < 0.05$, orange, $p < 0.01$, red, $p < 0.001$), and the analysis includes 234 water samples from all the stations. SSWWH: sea surface wind wave significant height.

	**1**	**2**	**3**	**4**	**5**	**6**	**7**	**8**	**9**	**10**	**11**	**12**
**SST (°C) (1)**	-	-0.07	0.20	0.59	0.11	-0.02	-0.08	-0.08	-0.41	0.37	0.47	-0.18
**Salinity (2)**		-	-0.22	0.33	-0.32	0.30	0.17	-0.61	0.61	0.19	-0.52	0.36
**Chl *a* (mg m^{-3}) (3)**			-	0.06	0.33	-0.09	0.15	0.23	-0.22	0.40	0.52	-0.21
**Babterial abundance (cells ml^{-1}) (4)**				-	-0.07	0.19	0.02	-0.29	-0.03	0.45	0.21	0.17
**PO_4^{3-} (µM) (5)**					-	-0.09	0.46	0.12	-0.22	0.32	0.21	-0.04
**NH_4^+ (µM) (6)**						-	0.18	-0.35	0.09	0.18	-0.23	0.23
**NO_3^- (µM) (7)**							-	-0.11	0.10	0.25	-0.01	-0.03
**Oxygen (mg l^{-1}) (8)**								-	-0.08	-0.02	0.29	-0.22
**Secchi depth (m) (9)**									-	0.07	-0.55	0.27
**Sediment bacteria (cells ml^{-1}) (10)**										-	0.21	0.19
**Relative abundance harmful cyanobacteria (11)**											-	-0.13
**SSWWH (m) (12)**												-

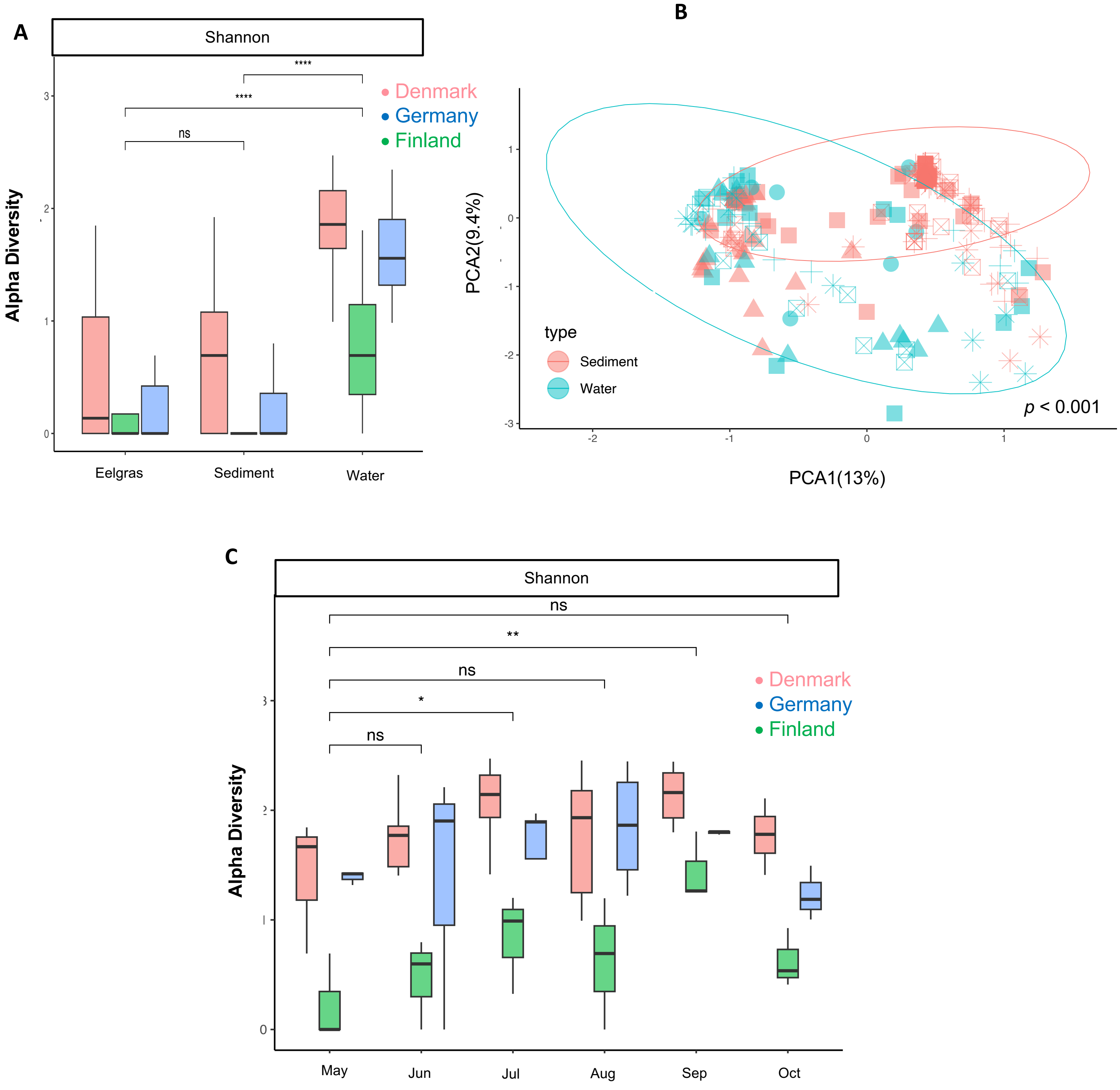
A
Shannon
Alpha Diversity
ns
****
****
Denmark
Germany
Finland
Eelgras
Sediment
Water
B
PCA2(9.4%)
PCA1(13%)
type
Sediment
Water
p < 0.001
C
Shannon
Alpha Diversity
ns
**
ns
*
ns
Denmark
Germany
Finland
May
Jun
Jul
Aug
Sep
Oct

Figure S1. *Vibrio* composition in the different compartments sampled, i.e., water, sediment, and eelgrass**. (A)** *Vibrio* spp. alpha diversity in water, sediment, and eelgrass. **B**) Principal component analysis (PCA) of the *Vibrio* spp. composition in waters and sediments. Eigen-values are 10.78 for PCA1 and 7.75 for PCA2. Reported p values were calculated by a PERMANOVA test, and circles enclose sample groups (sediment and water, n = 282). **C**) *Vibrio* spp. alpha diversity over time in the water columns of the three stations. In (**A**), values are the median of all values collected at the three substations from eelgrass, sediment, and water. In (**C**), values are the median of the replicates from the three substations at the specific time. The vertical lines indicate the position of the lower and upper quartiles. The data from two monthly sampling points in Denmark and Germany (only July and August) were combined to enable comparisons with the Finnish station. Asterisks indicate pairwise significant differences between the type of sample or month, (*$p < 0.05$, ** $p < 0.01$, ****, $p < 0.0001$), using a posthoc test (Wilcoxon) after a Kruskal-Wallis test.

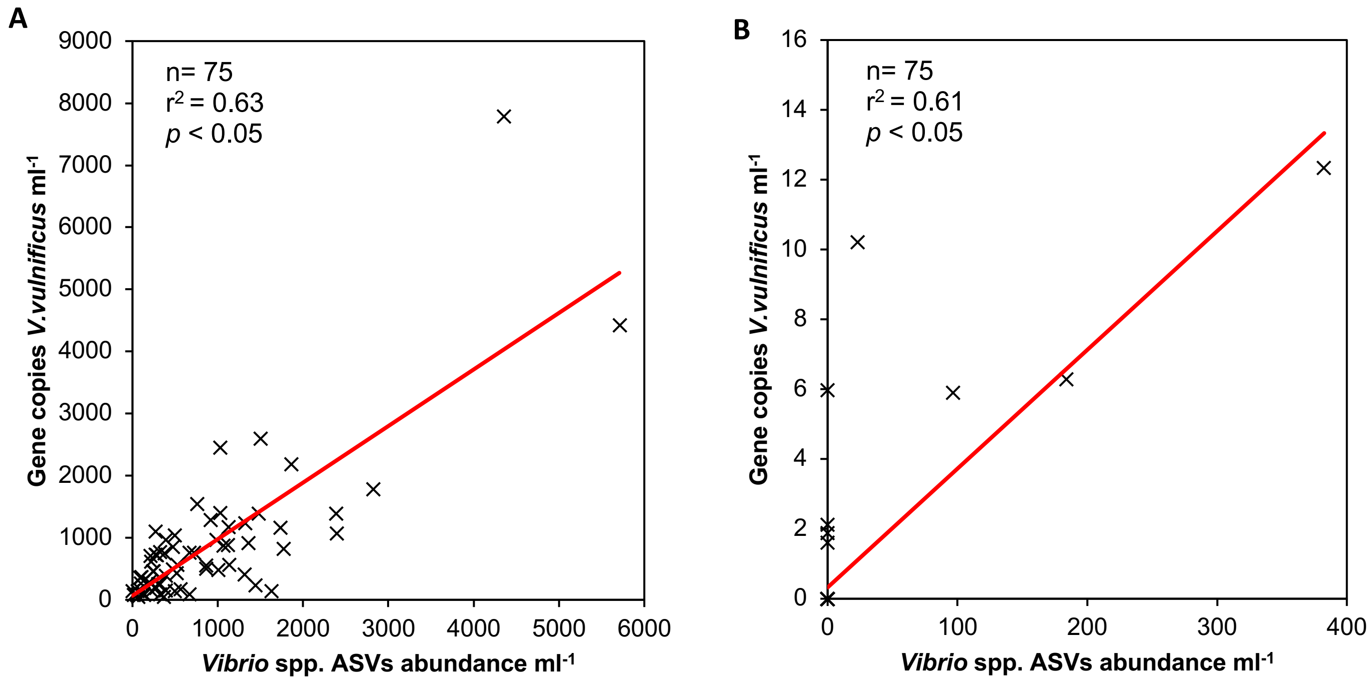

Figure S2. Regression scatter plots between *Vibrio* spp. and *V. vulnificus* ASVs abundance (obtained by multiplying the relative *Vibrio* ASVs frequencies with bacterial abundance in each sample) vs. the gene copies of *Vibrio* spp. (**A**) and *V. vulnificus* (**B**) quantified by ddPCRs. The triplicate ddPCR values were combined by calculating their average to enable comparisons with the pooled water samples that were sequenced.

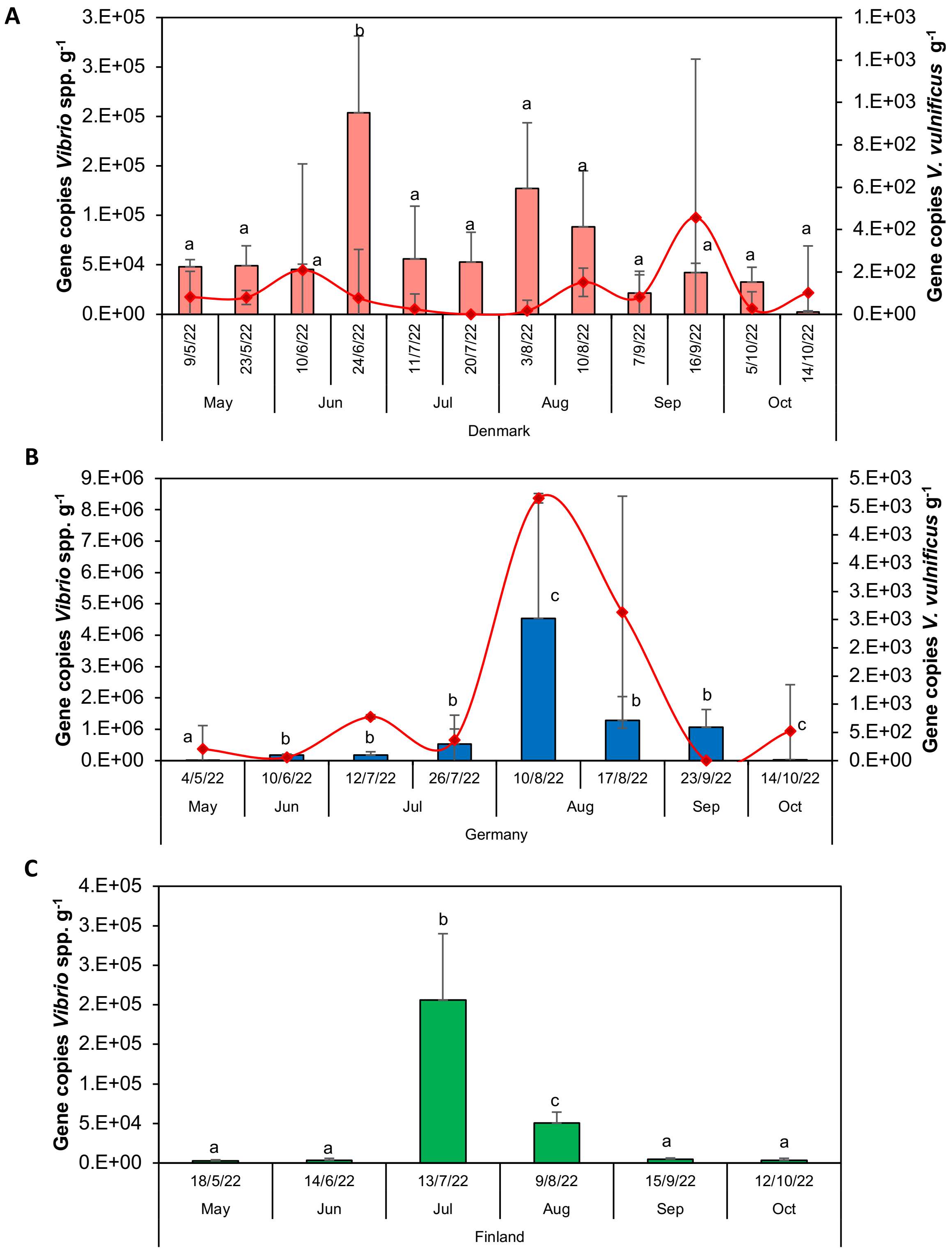

Figure S3. Temporal dynamics in the abundance of *Vibrio* spp. and *V. vulnificus* in sediment at the **A**) Danish, **B**) German, and **C**) Finish stations quantified by ddPCR. The values are the average ± the standard error between the replicates (n = 9). Letters indicate pairwise analysis among the variables (i.e., month) at each station using a posthoc test (Wilcoxon) after Kruskal-Wallis for *Vibrio* spp. Bars and red lines represent the gene copies of *Vibrio* spp. and *V. vulnificus*, respectively.

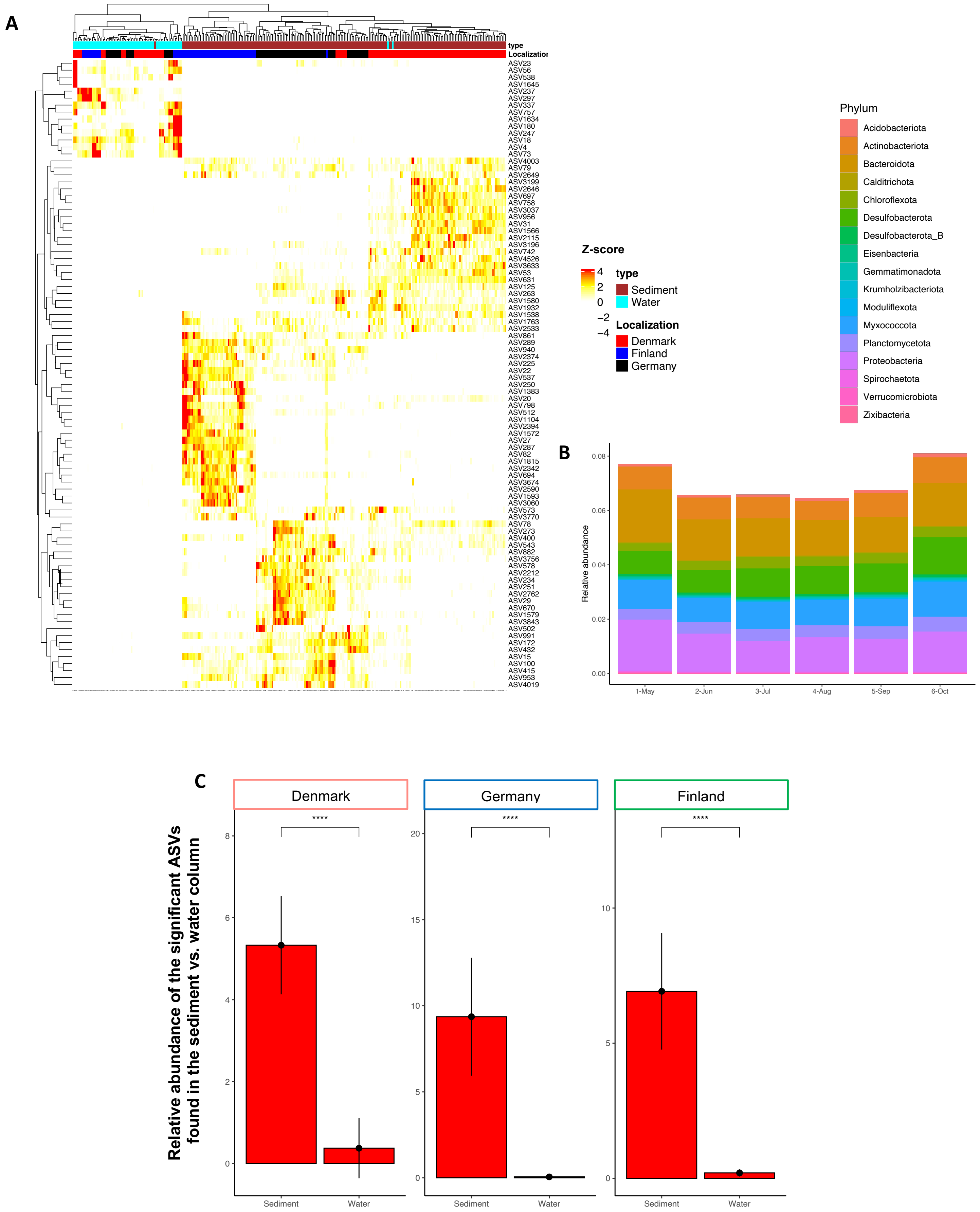
A
type
Localization
ASV23
ASV56
ASV538
ASV1645
ASV237
ASV297
ASV337
ASV757
ASV1634
ASV180
ASV247
ASV18
ASV4
ASV73
ASV4003
ASV79
ASV2649
ASV3199
ASV2646
ASV697
ASV758
ASV3037
ASV956
ASV31
ASV1566
ASV2115
ASV3196
ASV742
ASV4526
ASV3633
ASV53
ASV631
ASV125
ASV263
ASV1580
ASV1932
ASV1538
ASV1763
ASV2533
ASV861
ASV289
ASV940
ASV2374
ASV225
ASV22
ASV537
ASV250
ASV1383
ASV20
ASV798
ASV512
ASV1104
ASV2394
ASV1572
ASV27
ASV287
ASV82
ASV1815
ASV2342
ASV694
ASV3674
ASV2590
ASV1593
ASV3060
ASV573
ASV3770
ASV78
ASV273
ASV400
ASV543
ASV882
ASV3756
ASV578
ASV2212
ASV234
ASV251
ASV2762
ASV29
ASV670
ASV1579
ASV3843
ASV502
ASV991
ASV172
ASV432
ASV15
ASV100
ASV415
ASV953
ASV4019
Z-score
4
2
0
−2
−4
type
Sediment
Water
Localization
Denmark
Finland
Germany
Phylum
Acidobacteriota
Actinobacteriota
Bacteroidota
Calditrichota
Chloroflexota
Desulfobacterota
Desulfobacterota_B
Eisenbacteria
Gemmatimonadota
Krumholzibacteriota
Moduliflexota
Myxococcota
Planctomycetota
Proteobacteria
Spirochaetota
Verrucomicrobiota
Zixibacteria
B
Relative abundance
0.08
0.06
0.04
0.02
0.00
1-May
2-Jun
3-Jul
4-Aug
5-Sep
6-Oct
C
Relative abundance of the significant ASVs found in the sediment vs. water column
Denmark
Germany
Finland
****
****
****
8
6
4
2
0
20
15
10
5
0
10
5
0
Sediment
Water
Sediment
Water
Sediment
Water

Figure S4. ASVs identified as differentially abundant in the sediment utilized as indicators for sediment resuspension in the water column (used in Fig. 5). **A**) Heat map with cluster analysis of the most abundant, dominant, and significant ASVs identified by DESeq2 in the sediment (brown, n = 204) and water (blue cyan, n = 78) ($p < 0.01$). The heat map scale displays the row Z-score, i.e., the normalized taxa abundances (abundance of one ASV in one sample) - (mean abundance for that ASV))/(standard deviation of that ASV)). **B**) Phylum classification of the top ASVs identified as indicators for sediment resuspension. **C**) Comparison of the relative abundance of the ASVs detected by DESeq2 in water and sediment at each station. Values are the average ± the standard error between the replicates. Asterisks indicate pairwise significant differences between water and sediment (**** $p < 0.0001$), using a posthoc test (Wilcoxon) after a Kruskal-Wallis test.

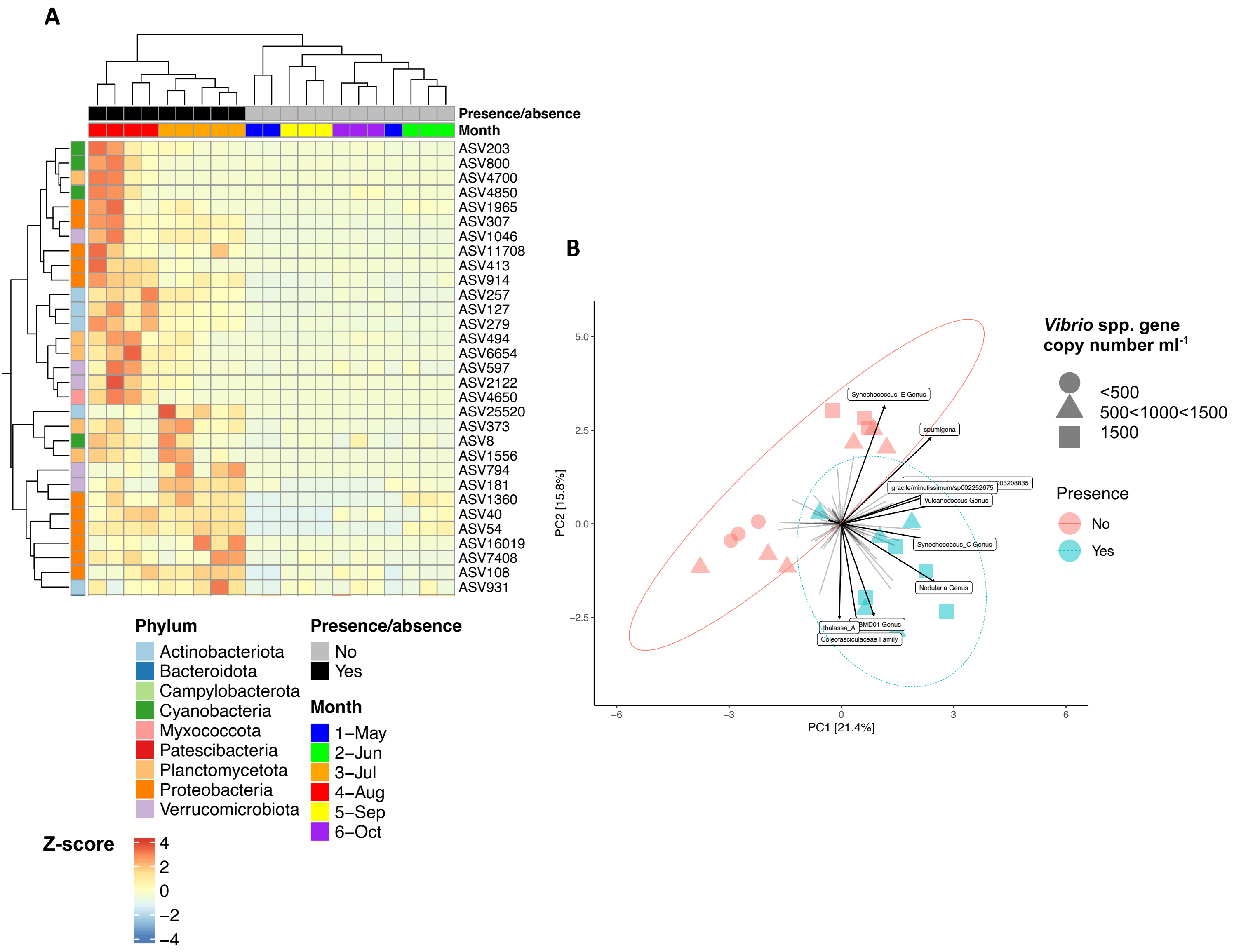
A
Presence/absence
Month
ASV203
ASV800
ASV4700
ASV4850
ASV1965
ASV307
ASV1046
ASV11708
ASV413
ASV914
ASV257
ASV127
ASV279
ASV494
ASV6654
ASV597
ASV2122
ASV4650
ASV25520
ASV373
ASV8
ASV1556
ASV794
ASV181
ASV1360
ASV40
ASV54
ASV16019
ASV7408
ASV108
ASV931
Phylum
Actinobacteriota
Bacteroidota
Campylobacterota
Cyanobacteria
Myxococcota
Patescibacteria
Planctomycetota
Proteobacteria
Verrucomicrobiota
Presence/absence
No
Yes
Month
1–May
2–Jun
3–Jul
4–Aug
5–Sep
6–Oct
Z-score
4
2
0
−2
−4
B
PC2 [15.8%]
5.0
2.5
0.0
−2.5
PC1 [21.4%]
−6
−3
0
3
6
Synechococcus_E Genus
spumigena
003208835
gracile/minutissimum/sp002252675
Vulcanococcus Genus
Synechococcus_C Genus
Nodularia Genus
BMD01 Genus
thalassa_A
Coleofasciculaceae Family
Vibrio spp. gene copy number ml-1
<500
500<1000<1500
1500
Presence
No
Yes

Figure S5. Dominating taxa when *V. vulnificus* was present. **A**) Heat map with cluster analysis of the top 30 most significant and dominant ASVs identified by DESeq2 as differentially abundant when *V. vulnificus* was present (black) or absent (grey) ($p < 0.01$). The heatmap scale displays the row Z-score. **B**) PCA of the cyanobacterial community according to the presence/absence of *V. vulnificus*. Eigen-values are 587.7 for PCA1 and 487.3 for PCA2. Arrows with cyanobacterial taxa represent the 10 most abundant taxa in the community. Reported p values were calculated by a PERMANOVA test performed with 999 permutations, and circles enclose sample groups. In **A** and **B**, "No" and "Yes" mean presence (n = 9) and absence (n = 12) of *V. vulnificus*. For these analyses, only the data from Germany was used, as *V. vulnificus* in the water column was only detected in this area. Clr-transformed data was used to perform the analyses in (**B**).
